# Introgression and repeated co‐option facilitated the recurrent emergence of C_4_ photosynthesis among close relatives

**DOI:** 10.1111/evo.13250

**Published:** 2017-04-28

**Authors:** Luke T. Dunning, Marjorie R. Lundgren, Jose J. Moreno‐Villena, Mary Namaganda, Erika J. Edwards, Patrik Nosil, Colin P. Osborne, Pascal‐Antoine Christin

**Affiliations:** ^1^ Department of Animal and Plant SciencesUniversity of SheffieldSheffieldS10 2TNUnited Kingdom; ^2^ Makerere UniversityKampalaUganda; ^3^ Department of Ecology and Evolutionary BiologyBrown UniversityProvidenceRhode Island02912

**Keywords:** Ancestral state, complex trait, co‐option, reticulate evolution, species tree

## Abstract

The origins of novel traits are often studied using species trees and modeling phenotypes as different states of the same character, an approach that cannot always distinguish multiple origins from fewer origins followed by reversals. We address this issue by studying the origins of C_4_ photosynthesis, an adaptation to warm and dry conditions, in the grass *Alloteropsis*. We dissect the C_4_ trait into its components, and show two independent origins of the C_4_ phenotype via different anatomical modifications, and the use of distinct sets of genes. Further, inference of enzyme adaptation suggests that one of the two groups encompasses two transitions to a full C_4_ state from a common ancestor with an intermediate phenotype that had some C_4_ anatomical and biochemical components. Molecular dating of C_4_ genes confirms the introgression of two key C_4_ components between species, while the inheritance of all others matches the species tree. The number of origins consequently varies among C_4_ components, a scenario that could not have been inferred from analyses of the species tree alone. Our results highlight the power of studying individual components of complex traits to reconstruct trajectories toward novel adaptations.

Inferences of transitions among character states along species phylogenies provide powerful tools to test specific hypotheses about the timing and rate of functional diversification, correlations among functional and ecological traits (e.g., Pagel 1999; Edwards et al. 2010; Danforth et al. 2013; Moreau and Bell 2013; McGuire et al. 2014; Halliday et al. 2016), and speciation rates (Rabosky et al. 2013; Cantalapiedra et al. 2017; Cooney et al. 2017). However, distinguishing between a single origin of a trait with subsequent losses versus multiple independent origins can be problematic (Whiting et al. 2002; Pagel 2004; Wiens et al. 2006; Gamble et. al. 2012), particularly when some character states affect the rates of speciation and/or extinction, when rates of transitions are high and asymmetrical, or variable among clades and through time (Maddison 2006; Goldberg and Igic 2008; Beaulieu et al. 2013; Igic and Busch 2013; King and Lee 2015). Indeed, transition rates might be higher in taxonomic groups possessing evolutionary precursors that increase the likelihood of evolving a specific trait (Blount et al. 2008, 2012; Marazzi et al. 2012; Christin et al. 2013a, 2015; Werner et al. 2014). This can lead to an unbalanced distribution of character states across the tree, with clusters forming in certain clades. However, a low rate of origins would lead to similar patterns if the rate of reversals is high (Wiens 1999; Danforth et al. 2003; Trueman et al. 2004; Pyron and Burbink 2014). Difficulties worsen if hybridization and introgression disconnect the history of underlying traits from the species tree (Pardo‐Diaz et al. 2012; Meier et al. 2017).

An alternative approach to analyzing the phenotypes as different character states is to decompose them into their constituent parts, then carefully analyze the evolution of each element independently to understand how the trait has been assembled or lost (Christin et al. 2010; Oliver et al. 2012; Niemiller et al. 2013; Kadereit et al. 2014). The use of distinct components can be interpreted as evidence for multiple origins, while reversals could leave a signature of the lost trait that can be detected when components are compared with those from species that never evolved it (Protas et al. 2006; Christin et al. 2010; Oliver et al. 2012; Niemiller et al. 2013). Identifying the mutations that underlie a trait further helps to distinguish shared origins and reversals (Igic et al. 2006; Shimizu et al. 2008; Niemiller et al. 2013; Meier et al. 2017). Evaluating the number of origins of each component of a complex trait would reconstruct the order of modifications that led to the trait of interest. This approach is applied here to the photosynthetic diversity exhibited within a five‐species taxonomic group.

C_4_ photosynthesis is a complex phenotype that improves the efficiency of carbon fixation in warm and dry conditions when compared to the ancestral C_3_ photosynthetic pathway (Sage et al. 2012; Atkinson et al. 2016). The C_4_ advantages are achieved by increasing the concentration of CO_2_ around Rubisco, the enzyme responsible for inorganic carbon fixation in the Calvin cycle of all photosynthetic organisms (von Caemmerer and Furbank 2003; Sage et al. 2012). To function, C_4_ photosynthesis requires the coordinated action of numerous anatomical and biochemical components that lead to the emergence of a novel biochemical pathway, usually across two types of cells; the mesophyll and bundle sheath cells (Hatch 1987; Prendergast et al. 1987; Gowik et al. 2011; GPWGII 2012; Bräutigam et al. 2014). Besides the increased expression of genes coopted for a C_4_ function, several other changes are known to occur during the evolution of C_4_ photosynthesis, including an expansion of bundle sheath tissue, a concentration of chloroplasts within it, and the adaptation of the enzymes to the new catalytic context (Fig. 1; Bläsing et al. 2000; von Caemmerer and Furbank 2003; McKown and Dengler 2007; Sage et al. 2012).

**Figure 1:**
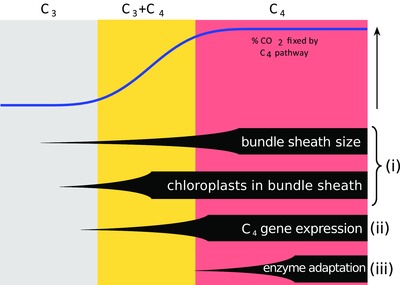
Schematic of expected changes during the transition from C_3_ to C_4_. The continuous variation in anatomical and biochemical components can be simplified using three phenotypic categories; C_3_ plants, C_3_+C_4_ intermediates, and C_4_ plants. A schematic indicates the proportion of atmospheric CO_2_ fixed by the C_4_ cycle, and the expected order of modifications is shown at the bottom for four categories of changes, with the number on the right indicating the section of our analyses where they are investigated.

Despite its apparent complexity, C_4_ photosynthesis evolved multiple times independently, and is present in distantly related groups of plants (Sinha and Kellogg 1996; Kellogg 1999; Sage et al. 2011). As with any complex trait, C_4_ photosynthesis likely evolved in incremental steps, via stages that are functionally intermediate and gradually increase carbon assimilation in warm and dry conditions (Fig. 1; Sage et al. 2012; Heckmann et al. 2013; Williams et al. 2013; Mallmann et al. 2014; Christin and Osborne 2014). An increase in bundle sheath size and the relocation of the chloroplasts/Rubisco to these cells can sustain a photorespiratory bypass (Hylton et al. 1988; Bräutigam and Gowik 2016). Subsequent increases in C_4_ enzyme abundances can generate a weak C_4_ cycle, which assimilates some of the atmospheric CO_2_, complementing the C_3_ cycle in C_3_+C_4_ plants (referred to as "type II C_3_‐C_4_ intermediates" in the specialized literature; Fig. 1; Heckmann et al. 2013; Mallmann et al. 2014). The transition to a full C_4_ state involves further increases of the bundle sheath tissue and gene expression, while selective pressures adapt the C_4_ enzymes for the new biochemical context (Fig. 1; Bläsing et al. 2000; McKown and Dengler 2007).

In the angiosperm phylogeny, C_4_ taxa form clusters, many of which have multiple C_4_ clades that are separated by non‐C_4_ branches (Sage et al. 2011; GPWGII 2012). Thus, establishing past photosynthetic transitions is difficult when photosynthetic type is modeled as a simple binary character (Ibrahim et al. 2009; Christin et al. 2010; Hancock and Edwards 2014; Bohley et al. 2015; Fisher et al. 2015; Washburn et al. 2015). Overall, nonhomology of key C_4_ components among some closely related C_4_ groups, including the cells, enzymes, and genes modified to generate the C_4_ pathway (Prendergast et al. 1987; Soros and Dengler 2001; Bräutigam et al. 2014; Lundgren et al. 2014; Wang et al. 2014), points to a predominance of C_4_ origins (Sinha and Kellogg 1996; Christin and Besnard 2009; Christin et al. 2010). However, the possibility of evolutionary reversals to a non‐C_4_ state is still debated (e.g., Kadereit et al. 2014; Bohley et al. 2015; Washburn et al. 2015). Furthermore, some components of the C_4_ phenotype (e.g., expansion of bundle sheaths and migration of chloroplasts; Fig. 1) may have evolved relatively few times, and have then been recurrently used for independent transitions to C_3_+C_4_ or C_4_ photosynthesis (Christin et al. 2011, 2013a).

One of the proposed candidates for an evolutionary reversal from C_4_ to C_3_ is in the grass genus *Alloteropsis* (Ibrahim et al. 2009). Within this genus, the species *Alloteropsis semialata* contains C_3_, C_3_+C_4_, and C_4_ genotypes (Ellis 1974; Brown 1975; Lundgren et al. 2016). In molecular phylogenies based on either plastid or nuclear markers, this species is sister to the C_4_ *Alloteropsis angusta*, and the two species form a monophyletic clade sister to the three remaining closely‐related C_4_ species: *Alloteropsis cimicina*, *A. paniculata*, and *A. papillosa* (Ibrahim et al. 2009; Christin et al. 2012; Olofsson et al. 2016). The C_4_ *A. semialata* and *A. cimicina* use different cell types for the segregation of C_4_ reactions (Renvoize 1987), which suggests independent realizations of C_4_ photosynthesis (Christin et al. 2010). However, the evolutionary origins of C_4_ biochemistry and the situation within the *A. angusta*/*A. semialata* group remain largely unexplored.

In this study, we focus on the genus *Alloteropsis* and its C_3_ outgroup, to test the competing hypotheses of multiple origins versus fewer origins followed by reversals, independently for each C_4_ component. A C_4_ phenotype generated via distinct cells, genes, or amino acid mutations would indicate independent origins. In contrast, a reversal may lead to a derived state that retains traces of its past C_4_ state when compared to the ancestral one (i.e., approximated by the C_3_ outgroup here). We combined different approaches to investigate different components of the complex C_4_ trait. (i) Focusing on anatomical characters, we evaluate the most likely number of episodes of movement of chloroplasts to the bundle sheath, and expansion of this tissue. (ii) Using transcriptome analyses to estimate gene expression, we then determine the most likely number of origins of a C_4_ cycle via the upregulation of known C_4_ photosynthetic genes. (iii) The number of episodes of enzyme adaptation for the C_4_ cycle is estimated using positive selection analyses, with scenarios corresponding to episodes of adaptation along different sets of branches. (iv) Finally, we compare divergence times across genes, to detect potential introgression of C_4_ components, as suggested within this genus for two C_4_ genes (Olofsson et al. 2016). Our multifaceted effort highlights the power of comparative analyses that directly consider genes and other components involved in the trait of interest, rather than modeling complex phenotypes as states of a single character. Using this approach, we show that recurrent origins of C_4_ photosynthesis in *Alloteropsi*s arose via a complex mixture of co‐option of traits increasing C_4_ accessibility, hybridization, and independent adaptation of the phenotype.

## Methods

### TAXON SAMPLING

The different datasets were obtained from plants grown under controlled conditions (See Supporting Information Methods 1.1 for detailed description of growth conditions), including one *Alloteropsis cimicina* (C_4_) accession, one *A. paniculata* (C_4_) accession, two *A. angusta* (C_4_) accessions, and up to 10 different *A. semialata* accessions collected from separate populations that encompass the global genetic and photosynthetic diversity of this species (one C_3_, two C_3_+C_4_ intermediates with a weak C_4_ cycle, and seven C_4_ accessions; Table S1; Lundgren et al. 2016). The over representation of C_4_ *A. semialata* accessions mirrors their natural abundance, with C_4_ accessions spread throughout Africa, Asia, and Australia, C_3_ accessions only reported in Southern Africa, and C_3_+C_4_ individuals restricted to central East Africa (Lundgren et al. 2015). We also make use of species representing the C_3_ sister group to *Alloteropsis* (*Panicum pygmaeum* and *Entolasia marginata*), previously identified using plastid markers (GPWGII 2012). Using the above taxa, we conduct four complementary sets of analyses, each providing insight into the origins or spread of distinct components of C_4_ in *Alloteropsis*.

### (i) COMPARING LEAF ANATOMIES AMONG PHOTOSYNTHETIC TYPES

Leaf cross‐sections were analyzed to identify the leaf compartment being used for the segregation of Rubisco and the modifications that increased the proportion of bundle sheath tissue in C_3_+C_4_ and C_4_ accessions. Co‐option of different tissues and distinct modifications among accessions would support independent origins, while a reversal should result in the leaves of C_3_ individuals having reverted to a state that retain traces of their past C_4_ state when compared to the ancestral condition (e.g., enlarged bundle sheath cells and/or chloroplasts in the bundle sheath).

We generated new anatomical data for nine *A. semialata* accessions and *A. angusta* (Table S2), which supplemented previously published anatomical data for *E. marginata*, *P. pygmaeum*, *A. cimicina*, and *A. paniculata* (Christin et al. 2013a). Images of *A. semialata* and *A. angusta* leaves in cross‐section were obtained by fixing the center portion of a mature leaf blade in 4:1 ethanol:acetic acid, embedding them in methacrylate embedding resin (Technovit 7100, Heraeus Kulzer GmbH, Wehrhein, Germany), sectioning on a manual rotary microtome (Leica Biosystems, Newcastle, U.K.), staining with Toluidine Blue O (Sigma‐Aldrich, St. Louis, MO), then photographing them with a camera mounted atop a microscope (Olympus DP71 and BX51, respectively, Olympus, Hamburg, Germany), as described in Lundgren et al. (2016).

All species used in this study have two bundle sheath layers, differentiated as inner and outer bundle sheaths, which create concentric circles around each vein (Fig. S1). The sheath co‐opted for the segregation of Rubisco was identified by a concentration of chloroplasts producing starch. We also recorded the presence of minor veins, and measured the following traits on one cross‐sectional image per accession, as described in Christin et al. (2013a), using ImageJ software (Schneider et al. 2012): the interveinal distance (IVD; the average distance between centers of consecutive veins), the number of mediolateral mesophyll cells between veins, the average width of all outer and inner bundle sheath cells within a leaf segment, and the ratio of outer to inner bundle sheath cell widths (OS:IS). One leaf cross‐section was used per accession, with previous work showing the traits we are measuring exhibit little variation within populations (Lundgren et al. 2016).

### (ii) COMPARING GENE EXPRESSION PROFILES AMONG PHOTOSYNTHETIC TYPES

We use RNA‐Seq to identify the genes co‐opted by the different accessions performing a C_4_ cycle, as those encoding C_4_‐related enzymes that reach high abundance in C_4_ leaves. Variation in the co‐opted loci would support multiple origins of a weak C_4_ cycle, while a reversal might lead to high expression of C_4_‐related genes in individuals without a C_4_ cycle or loss of functions of genes previously used for the C_4_ cycle.

For RNA‐Seq, we sampled the highly photosynthetically active distal halves of fully expanded new leaves and fresh roots midway into the photoperiod, which were subsequently flash frozen. Two different photoperiods (i.e., 10 and 14 h) were used to ensure that the identification of the most highly expressed genes did not differ among light regimes. Data from root libraries were only used in this study for transcriptome assembly, while all leaf samples were used for both assembly and quantification of transcript abundances. For a full list of individuals, conditions, and tissues sampled see Table S3.

Total RNA was extracted, Illumina TruSeq libraries generated, and sequencing performed using standard laboratory procedures, and transcriptomes were assembled using available pipelines (see Supporting Information Methods 1.2 for a detailed description of RNA‐Seq protocol and assembly statistics). For each assembled contig, the transcript abundance was calculated as reads per million of mapped reads (rpm). Using a previously developed phylogenetic annotation pipeline (Christin et al. 2013b, 2015), the transcript abundance was then calculated for each gene lineage encoding C_4_‐related enzymes. For each gene family, all sequences descending from a single gene in the common ancestor of grasses via speciation and/or duplication were considered as the same gene lineage (i.e., these are grass co‐orthologs). These groups include potential lineage specific paralogs (i.e., also known as inparalogs). When different *Alloteropsis* genes were identified within the same group of co‐orthologs through detailed phylogenetic analyses, the abundance of each group was estimated independently. In *Alloteropsis*, this is the case only for genes previously shown to have been acquired laterally from distantly related C_4_ lineages (Christin et al. 2012; see Results). In short, the reference datasets, composed of *Arabidopsis thaliana* coding sequences annotated as encoding C_4_‐related enzymes, and homolog sequences from other completely sequenced plants including five grasses, were retrieved from Christin et al. (2013b; 2015), or generated following the same approach for additional C_4_‐related enzymes identified in more recent studies (Mallmann et al. 2014; Li et al. 2015; Fig. S2). Contigs with similar sequences from the transcriptomes generated here were identified using BLASTn, with a minimal *e*‐value of 0.01, and a minimal matching length of 50 bp. Only the portion of the contig matching the references was considered to remove UTRs, potential introns, and other very variable segments. Each sequence retrieved this way was then aligned independently to the reference dataset using Muscle (Edgar 2004), and a phylogenetic tree was inferred using Phyml (Guindon and Gascuel 2003) with a GTR+G+I model, a model that fits the vast majority of genes (e.g., Fisher et al. 2016) and is appropriate to infer a large number of trees. Phylogenetic trees were automatically screened, and each contig was assigned to the previously identified gene lineages in which it was nested. The sum of rpm values of all transcriptome contigs assigned to the same gene lineage produced transcript abundance per group of grass co‐orthologs or distinct genes within these groups, which were subsequently transformed into rpm per kilobase (rpkm) values. Rpkm values were then compared among accessions to identify similarities and differences in the expression of C_4_ photosynthetic genes.

### (iii) GENE TREES AND DETECTION OF ENZYME ADAPTATION FOR C_4_ PHOTOSYNTHESIS

Phylogenetic trees were inferred for C_4_‐related genes that were highly abundant in the leaf transcriptomes of at least two C_4_ *Alloteropsis* samples (identified from transcriptome data; see Results) and their co‐orthologs in other C_3_ and C_4_ grasses (see Supporting Information Methods 1.3 for a detailed description of phylogeny construction). The inferred gene trees were used to verify that C_4_‐related genes were placed as expected based on the species tree, as opposed to a position suggesting an acquisition from distant C_4_ relatives. In addition, the gene tree topologies were used for positive selection analyses to detect traces of past episodes of enzyme adaptation for the new catalytic context after the initial emergence of a C_4_ cycle (Fig. 1; Blasing et al. 2000; Christin et al. 2007; Besnard et al. 2009; Wang et al. 2009; Heckmann et al. 2013; Mallmann et al. 2014; Huang et al. 2017). Positive selection on branches leading to each C_4_ group would support independent transitions to a full C_4_ cycle via enzyme adaptation, while an early origin followed by a reversal should result in positive selection in the common ancestor of all C_4_ accessions and possibly in the lineages that reversed back to the previous state.

For each set of genes encoding core C_4_ enzymes in at least two *Alloteropsis* accessions, identified via transcriptome analyses, we optimized several codon models (site and branch‐site models) to test for adaptive evolution using codeml as implemented in PAML (Yang 2007). The best‐fit model was identified among those that assume (0) no positive selection (M1a null model), and the branch‐site models that assume shifts in selection pressure, either to relaxed selection (model BSA) or to positive section (model BSA1), at the base of: (1) *Alloteropsis* (one round of enzyme adaptation), (2) both *A. cimicina* and *A. angusta* + *A. semialata* (two rounds of enzyme adaptation), and (3) *A. cimicina*, *A. angusta*, and *A. semialata* (three independent episodes of enzyme adaptation). Foreground branches for all models were specified as the branch leading to the identified node plus all descending branches (i.e., using a "$" sign as opposed to a "#"). Models involving positive selection in only one of the C_4_ lineages were also considered (see Supporting Information Methods 1.3 for additional details of positive selection analysis). For each gene lineage, the best‐fit model was identified based on the corrected Akaike information criterion (AICc), selecting the model with the lowest AICc after checking that its ΔAICc score was at least 5.22 units below that of the M1a null model. An ΔAICc score = 5.22 corresponds to a *P*‐value threshold of 0.01 for a likelihood ratio test comparing these two models using 2 degrees of freedom (df). C_4_ species other than *Alloteropsis* were removed prior to analysis to avoid an influence of positive selection in these taxa affecting our conclusion. Analyses were repeated using only codons with fixed nucleotides within each lineage (i.e., *A. angusta*, C_3_ *A. semialata*, C_3_+C_4_ *A. semialata*, and C_4_ *A. semialat*a), to verify that short terminal branches with unfixed mutations did not significantly inflate the dN/dS ratio, and therefore alter our conclusion. Finally, to assess the effect of gene tree topology on our conclusions, we repeated the positive selection analyses using 100 bootstrap pseudoreplicate topologies.

### (iv) DATING THE DIVERGENCE OF ADAPTIVE LOCI TO IDENTIFY INTROGRESSION

To determine whether introgression has spread C_4_ adaptations among species, we performed molecular dating of markers from across the transcriptomes, including those used for C_4_ by at least two *Alloteropsis* accession and their paralogs. The divergence times between species estimated from introgressed genes are expected to be younger than those estimated from other genes (e.g., Smith & Kronforst 2013; Li et al. 2014; Marcussen et al. 2014; Li et al. 2016), resulting either in outliers (if few genes are introgressed) or a multimodal distribution of ages (if many genes are introgressed).

Groups of genes descending from a single gene in the common ancestor of Panicoideae (Panicoideae co‐orthologs), the grass subfamily that includes *Alloteropsis*, were identified through phylogenetic analyses of our transcriptomes and completely sequenced genomes that were publicly available. Our automated pipeline started with gene families previously inferred for eight plant genomes (homologs: i.e., all the paralogs and orthologs; Vilella et al. 2009), including two Panicoideae grasses (*Setaria italica* and *Sorghum bicolor*), two non‐Panicoideae grasses (*Brachypodium distachyon* and *Oryza sativa*), and four nongrass species (*Amborella trichopoda, A. thaliana*, *Populus trichocarpa*, and *Selaginella moellendorffii*). To ensure accurate annotation, we restricted the analysis to gene families that included at least one *A. thaliana* sequence. The coding sequences (CDS) from the above genomes were then used to identify similar sequences in our transcriptomes using BLASTn with a minimum alignment length of 500 bp.

Stringent alignment and filtering methods were used to ensure reliable alignments of the above sequences for each gene family for phylogenetic inference (see Supporting Information Methods 1.4 for full details). In total, 2,797 1:1 Panicoideae co‐ortholog datasets were used for subsequent molecular dating, as implemented in Beast version 1.5.4 (Drummond and Rambaut 2007). For each dataset, divergence times were estimated based on third codon positions, to decrease the risk of selective pressures biasing the outputs. A log‐normal relaxed clock was used, with a GTR+G+I substitution model, and a constant coalescent prior. The *Sorghum* sequence was selected as the outgroup and the root of the tree was fixed to 31 Ma (using a normal distribution with a SD of 0.0001), based on estimates from Christin et al. (2014). There is uncertainty around this date, and the low species sampling used here probably leads to overestimation of both divergence times and confidence intervals, but the use of consistent sampling and calibration points among markers allows for the comparison of relative (rather than absolute) ages, which is the point of these analyses. Each Beast analysis was run for 2,000,000 generations, sampling a tree every 1,000 generations after a burn‐in period of 1,000,000. For nodes of interest, divergence times were extracted from the posterior distribution as medians.

Divergence times were also estimated for key genes used for C_4_ photosynthesis in *Alloteropsis* (identified based on transcriptomes; see Results), using the same parameters. To guarantee a consistent species sampling, the taxa included in the transcriptome‐wide analyses were retrieved from manually curated alignments for C_4_‐specific genes as well as other groups of orthologs from the same gene families, obtained as described above for C_4_‐specific forms. In addition, plastid genomes for the same species were retrieved from Lundgren et al. (2015), and reanalyzed with the same parameters. For each of these datasets, the median, 95% CI, and 0.25 and 0.75 quantiles were extracted from the posterior distribution, using the R package APE (Paradis et al. 2004).

## Results

### (i) DIFFERENT REALIZATIONS of C_4_ LEAF ANATOMY IN *A. CIMICINA* AND *A. SEMIALATA*/*A. angusta*

Grasses ancestrally possess two concentric rings of bundle sheath cells and either can be co‐opted for C_4_ photosynthesis (Brown 1975; Lundgren et al. 2014). The closely related C_4_ *A. cimicina* and *A. paniculata* co‐opted the outer bundle sheath for Rubisco segregation, as evidenced by the proliferation of chloroplasts in this tissue (Fig. S1; Table S2). In these species, the overall proportion of outer bundle sheath tissue within the leaf is increased via enlarged outer bundle sheath cells. Indeed, the outer sheath is 7.8‐fold larger than the inner sheath in C_4_ *A. cimicina* and *A. paniculata*, compared to a 1.2‐ to 0.6‐fold differences in C_4_ *A. semialata* and *A. angusta* (Table S2). This contrasts strongly with the anatomy of the C_4_ *A. semialata* and *A. angusta* (Fig. S1). Both of these species use the inner bundle sheath for Rubisco segregation and increase the overall proportion of this tissue via the proliferation of minor veins, and enlargement of the inner sheath cell size (Fig. S1; Table S2).

Staining by Toluidine Blue O indicates some starch production occurs in the inner bundle sheaths of both the C_3_ and C_3_+C_4_ *A. semialata* (Fig. S1), which implies some Rubisco activity in these cells, confirming previous reports (Ueno and Sentoku 2006; Lundgren et al. 2016). The absence of minor veins in the C_3_ and C_3_+C_4_ *A. semialata* results in a larger proportion of mesophyll compared to C_4_ *A. semialata* (Table S2; Fig. S1). In the C_3_ and C_3_+C_4_ *A. semialata*, the outer bundle sheath is slightly larger than the inner one (1.2‐ to 1.8‐fold; Table S2), while the C_3_ outgroup species *P. pygmaeum* and *E. marginata* have outer bundle sheaths that are considerably larger than their small inner sheaths (4.5‐ and 5.3‐fold; Fig. S1; Table S2).

**Figure 2:**
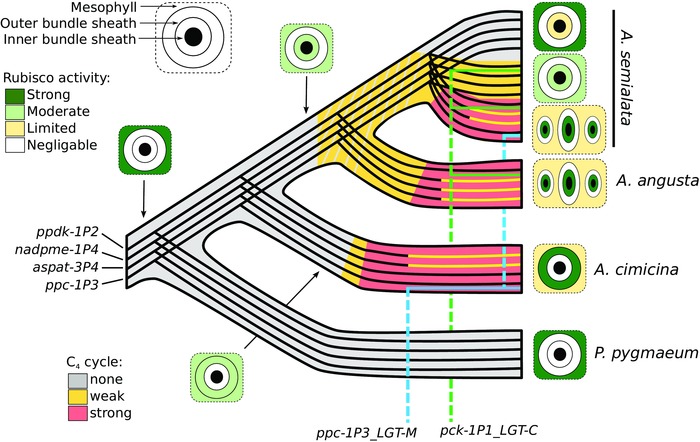
Inferred transitions among C_4_ components. A schematic phylogenetic tree is presented, based on previous genome‐wide analyses (Lundgren et al. 2015; Olofsson et al. 2016). Individual lines represent the transmission of individual genes within the species complex. For each of the four genes subject to C_4_‐related selection, episodes of positive selection are indicated by changes to yellow. Other lines track the spread of two genes that were originally laterally‐acquired from distant relatives, and have subsequently been introgressed among *Alloteropsis* species. The inferred phenotype is represented by the background colour, in grey for C_3_, in yellow for C_3_+C_4_, and in red for C_4_. The grey hatching indicates uncertainty about the ancestral state. A simplified version of leaf anatomy is represented, for extant taxa and some hypothetical ancestors (see Fig. S1 for details of leaf anatomy of extant accessions).

In summary, our comparative studies of leaf anatomy indicate that the C_4_ *A. cimicina* and *A. semialata*/*A. angusta* use different tissues for Rubisco segregation and achieve high bundle sheath proportions via distinct modifications, supporting independent origins of C_4_ anatomical components in these two groups. Some Rubisco activity is suggested in the inner sheath of the C_3_ *A. semialata*, which supports an early origin migration of chloroplasts to this tissue (Fig. 2). In addition, a slight enlargement of the inner sheath, absent in the C_3_ outgroup, is common to all non‐C_4_ *A. semialata*.

### (ii) *A.CIMICINA* USES DIFFERENT ENZYMES AND GENES FOR C_4_ BIOCHEMISTRY THAN *A. SEMIALATA/A. ANGUSTA*

**Figure 3:**
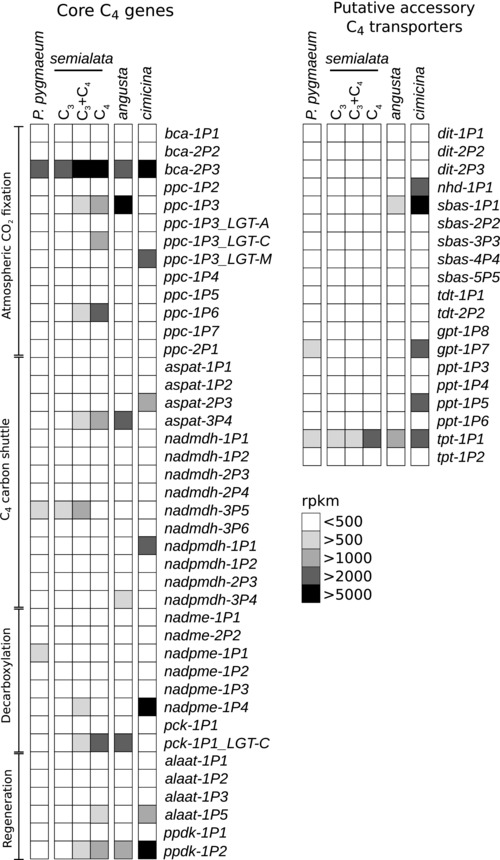
Expression of C_4_‐related enzymes in *Alloteropsis*. For each gene encoding a C_4_‐related enzyme, the shade indicates the category of transcript abundance, using averages per group. For raw values, see Table S4. Note that *ppc* abundance varies among C_4_ accessions of *A. semialata* (Fig. 4). The enzymes involved in core C_4_ reactions (left column) are grouped by functional property, and gene names are written in italics on the right of the expression values.

**Figure 4:**
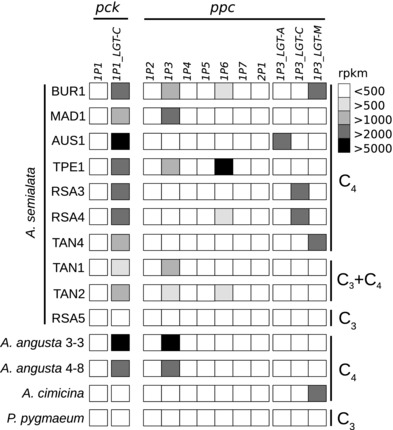
Leaf abundance of *pck* and *ppc* genes in the different accessions. The shade indicates the relative expression (in rpkm) in the different accessions. For each accession, the averages are used. For raw values, see Table S4.

All *Alloteropsis* C_3_+C_4_ and C_4_ accessions have high expression abundance in their leaves of co‐orthologs encoding phosphoenolpyruvate carboxylase (PEPC), the enzyme used for the initial fixation of atmospheric carbon into organic compounds in C_4_ plants. However, the gene lineage most highly expressed varies among accessions (Figs. 3 and 4). The close relationships between some of the genes for PEPC and one for phosphoenolpyruvate carboxykinase (PCK) isolated from *Alloteropsis* and those of distantly related C_4_ species was confirmed by our phylogenetic analyses (Figs. S3 and S4), supporting the previous conclusion that these genes were acquired by *Alloteropsis* via lateral gene transfer (LGT; Christin et al. 2012). Based on the read abundance, *A. cimicina* uses *ppc‐1P3_LGT‐M*, while *A. angusta* uses *ppc‐1P3* (Fig. 4). There is variation within *A. semialata*, with C_3_+C_4_ and C_4_ accessions using either one or a combination of several gene lineages several gene lineages (Fig. 4).

From the expression profiles (Fig. 3), the carbon shuttle of *A. cimicina* relies on enzymes and transporters associated with the most common form of C_4_ photosynthesis (NADP‐malic enzyme type; Gowik et al. 2011; Bräutigam et al. 2014; Mallman et al. 2014). This expression profile differs markedly from that observed in the C_4_ *A. semialata* and *A. angusta* accessions. These two species mainly use the PCK decarboxylating enzyme, through the high expression of the same gene (*pck‐1P1_LGT‐C*; Fig. 4). There is little evidence in these species for an involvement of the auxiliary transporters observed in *A. cimicina* (Fig. 3; Table S4), and some of the core enzymes are not shared by *A. cimicina* and *A. semialata*/*A. angusta* (Fig. 3). Furthermore, even when the same enzyme family is used, it is not necessarily encoded by the same locus (e.g., *A. cimicina* expresses *aspat‐2P3* and *A. semialata*/*A. angusta* express *aspat‐3P4*; Fig. 3).

The transcriptomes of the C_3_+C_4_ *A. semialata* show elevated levels of some of the genes used by the C_4_ *A. semialata*, with a slightly higher abundance of those encoding the NADP‐malic enzyme (*nadpme‐1P4*; Fig. 3; Table S4). In terms of the expression levels of genes encoding C_4_‐related enzymes, the transcriptome of the C_3_ *A. semialata* is not markedly different from that of the C_3_ outgroup *P. pygmaeum* (Fig. 3; Table S4).

Our comparative transcriptomics therefore indicate that *A. cimicina* uses different genes and different enzymes for the C_4_ pathway than *A. semialata*/*A. angusta*, suggesting multiple origins of the C_4_ cycle (Fig. 2). The only C_4_‐related genes used by some C_4_ *Alloteropsis* that are abundant in the C_3_ *A. semialata* (*bca‐2P3* and *tpt‐1P1*) are also highly expressed in the C_3_ outgroup and in other distantly related C_3_ taxa (Fig. 3; Külahoglu et al. 2014; Ding et al. 2015), indicating that high levels in leaves is not specific to our group of species. For the C_4_‐related genes used by the C_4_ *Alloteropsis*, but not abundant in the outgroup, there is no evidence for high expression or pseudogenization in the C_3_ *A. semialata*. Evidence is thus lacking that the C_3_ *A. semialata* represent a reversal from an ancestor with a C_4_ cycle.

### (iii) INDEPENDENT EPISODES OF C_4_‐RELATED POSITIVE SELECTION IN EACH C_4_ SPECIES

**Figure 5:**
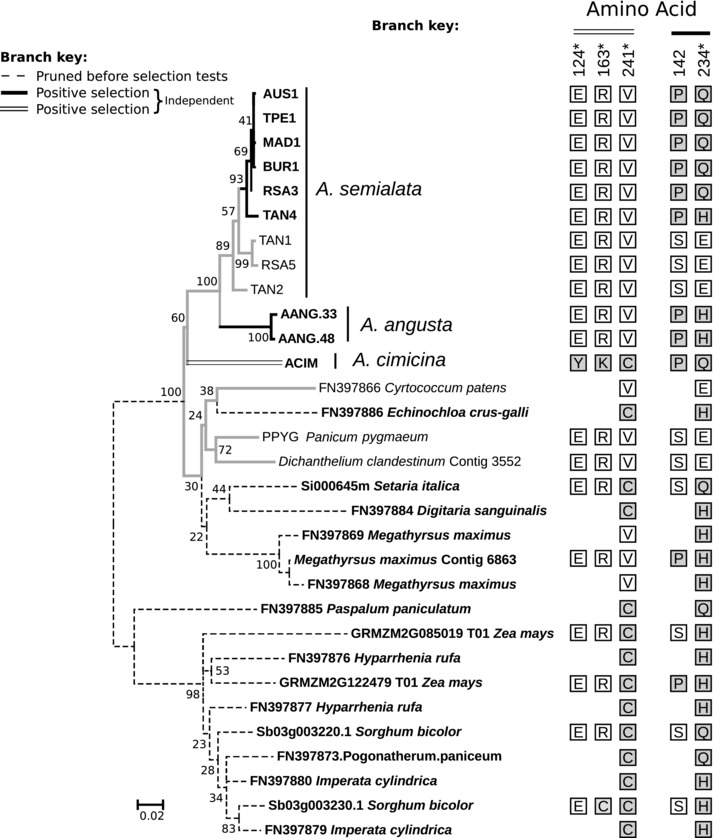
Evolution of *nadpme‐1P4* genes in *Alloteropsis* and other Panicoideae. This phylogenetic tree was inferred on 3^rd^ positions of codons of *nadpme‐1P4* genes of Panicoideae. Bootstrap values are indicated near branches. Names of C_4_ accessions are in bold. Amino acid at positions under positive selection are indicated on the right, with those associated with C_4_ accessions in gray. Positions are indicated on the top, based on *Sorghum* gene Sb03g003220.1. Amino acid positions with a posterior probability >0.90 of being under positive selection are indicated on the right, asterisks indicate positions with a posterior probability >0.95.

The codon models do not support positive selection on any genes involved in C_4_ photosynthesis at the base of *Alloteropsis* or along the branch leading to the *A. angusta*/*A. semialata* group (Table 1). In two cases (*nadpme‐1P4* and *ppdk‐1P2*), analyses including all *Alloteropsis* accessions clearly point to changes in selective pressures specifically in the branch leading to *A. cimicina* (Table 1; Fig. S5). No evidence of positive selection was found for the two other genes analyzed on the three *Alloteropsis* species (*aspat‐2P3* and *alaat‐1P5*; Table 1). When testing for selection only in the *A. angusta*/*A. semialata* clade, no positive selection was found on *ppdk‐1P2*, while positive selection on *ppc‐1P3* was identified only on the branch leading to *A. angusta* (Table 2). For the two other genes (*nadpme‐1P4* and *aspat‐3P4*), the model that assumes positive selection after the split of the two species was favored (Table 2). A majority of the amino acid sites identified as under positive selection by the Bayes Empirical Bayes analysis overlapped with those previously identified in other C_4_ taxa (e.g., site 241 in *nadpme‐1P4;* Fig. 5; Christin et al. 2009), or were shared with other C_4_ species in our phylogenies (e.g., Fig. 5), supporting their link to C_4_ photosynthesis. For *aspat‐3P4*, more amino acid substitutions were fixed in *A. angusta* than in *A. semialata*. This variation among *A. semialata* C_4_ accessions indicates repeated bouts of positive selection during the diversification of this species (Fig. S6). Conclusions based on the selection tests were also supported using only codons with fixed nucleotides within a lineage (i.e., photosynthetic types in *A. semialata*, and *A. angusta*), with the exception of *nadpme‐1P4* for which no positive selection was inferred after removing the unfixed codons (Tables S5 and S6). Furthermore, gene tree topology had no effect on our conclusions, since all bootstrap replicates supported the same model, with the exception of 2% of *nadpme*‐*1P4* bootstrap replicates (Tables S7 and S8).

**Table 1:** Results of positive selection analyses inferring the episodes of enzymatic adaptation in *Alloteropsis* 1

			One origin		Two origins		Three origins		Only *A. cimicina*	
Gene	Number of sequences	Site model M1a	BSA	BSA1	BSA	BSA1	BSA	BSA1	BSA	BSA1
*aspat‐2P3*	14	0.00*	4.02	4.02	4.02	4.02	3.94	4.02	4.00	4.00
*nadpme‐1P4*	15	35.07	30.44	27.26	26.34	24.28	19.52	13.31	3.34	0.00*
*ppdk‐1P2*	15	29.45	32.00	26.35	32.31	27.17	26.37	23.37	3.55	0.00*
*alaat‐1P5*	14	0.00*	1.74	1.74	2.03	2.03	0.69	0.69	4.02	4.02

^1^The ΔAICc values compared to the best‐fit model for that gene are shown. The most appropriate model is indicated with an asterisk, with the null model (M1a) only rejected if the ΔAICc was at least 5.22 (equivalent to a *P*‐value of 0.01 with a likelihood ratio test with df = 2). Two branch‐site models were used to test for a relaxation of purifying selection (BSA), and potential positive selection (BSA1).

**Table 2:** Results of positive selection analyses inferring the episodes of enzymatic adaption in the *A. angusta/A. semialata* clade^1^

			One origin		Two origins		Only *A. angusta*	
Gene	Number of sequences	Site model M1a	BSA	BSA1	BSA	BSA1	BSA	BSA1
*aspat‐3P4*	13	12.33	10.20	6.70	6.37	0.00*	5.45	5.29
*nadpme‐1P4*	14	10.19	14.19	9.66	13.52	0.00*	14.18	14.18
*ppc‐1P3*	9	72.43	66.62	66.58	11.70	9.85	5.66	0.00*
*ppdk‐1P2*	14	0.00*	4.01	4.01	4.01	4.01	3.91	3.91

^1^The ΔAICc values compared to the best‐fit model for that gene are shown. The most appropriate model is indicated with an asterisk, with the null model (M1a) only rejected if the ΔAICc was at least 5.22 (equivalent to a *P*‐value of 0.01, with a likelihood ratio test with df = 2). Two branch‐site models were used to test for a relaxation of purifying selection (BSA), and potential positive selection (BSA1).

Overall, our positive selection tests point to independent episodes of enzyme adaptation for the C_4_ context in each of the C_4_ groups (Fig. 2). None of the models that included adaptive evolution on branches leading to C_3_ and/or C_3_+C_4_ *A. semialata* were favored, suggesting no evolutionary loss of a full C_4_ cycle.

### (iv) GENES FOR PEPC AND PCK WERE SPREAD ACROSS SPECIES BOUNDARIES

**Figure 6:**
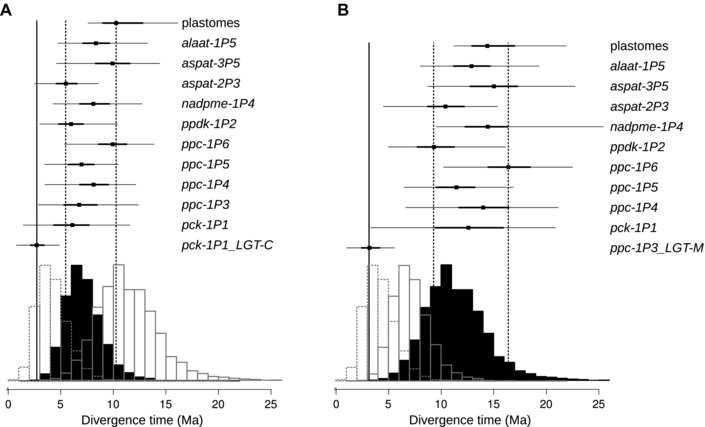
Estimates of divergence times. On the top, divergence times are shown for selected nuclear genes and plastomes for **A)** the split of *A. angusta* and *A. semialata* and **B)** the crown of *Alloteropsis*. For each marker, the median of the estimates is indicated by a square, with thick bars connecting the 25 and 75 percentiles and thin bars connect the 2.5 and 97.5 percentiles. The distribution of medians for the crown of *A. semialata* (left), the split of *A. angusta* and *A. semialata* (middle), and the crown of *Alloteropsis* (right) over 2,797 markers extracted from the transcriptomes is shown at the bottom. The scale is given in million years ago (Ma).

The 2,797 groups of orthologs extracted from genomes and transcriptomes led to a wide range of estimated divergence times, with 95% of the medians falling between 6.51 and 17.92 Ma for the crown of *Alloteropsis*, and between 4.17 and 11.27 Ma for the split of *A. semialata* and *A. angusta* (Fig. 6). The peak of values (i.e., 50% of the points) ranged between 9.38 and 13.07 Ma for the crown of *Alloteropsis* and 5.93 and 8.18 Ma for the split of *A. semialata* and *A. angusta* (Fig. 6). Finally, 95% of the markers estimated the crown of *A. semialata* between 1.88 and 7.77 Ma, with a peak between 3.12 and 5.07 Ma (Fig. 6). Note that monophyly of the groups was not enforced, and various combinations of *A. semialata* accessions were included across markers, contributing to the observed variation.

Most of the C_4_‐related genes, as well as the plastomes, provided age estimates ranging from 5.54 to 10.32 Ma for the split of *A. semialata* and *A. angusta*, which matches the distribution of estimates from the transcriptome‐wide data (Fig. 6A), and indicates their transmission followed the species tree. The only exception is the gene *pck‐1P1_LGT‐C*, for which the last common ancestor of *A. semialata* and *A. angusta* was estimated at 2.77 Ma (Fig. 6A), which is smaller than all but four of the 2,797 estimates from the transcriptome‐wide markers. While the confidence intervals of the estimate for this gene do overlap with those of almost all other markers, this estimate matches more closely the diversification of *A. semialat*a accessions (Fig. 6A).

The different markers selected for detailed analyses similarly yielded estimates for the crown of *Alloteropsis* matching those obtained from transcriptome‐wide data, between 9.38 and 16.46 Ma (Fig. 6B). The only exception is the gene *ppc‐1P3_LGT‐M*, for which the last common ancestor of *A. cimicina* and *A. semialata* is estimated at 3.25 Ma (Fig. 6B), which is smaller than all estimates based on markers extracted from the transcriptomes. The 95% CI of the divergence estimate based on this gene does not overlap with many of those based on other markers, and again matches closely with the diversification of *A. semialat*a accessions (Fig. 6B).

Overall, our dating analyses support an introgression of these two genes among *Alloteropsi*s species after their divergence, while the other genes were transmitted following the species tree (Fig. 2).

## Discussion

### TWO INDEPENDENT TRANSITIONS FROM C_3_ TO C_4_

The earliest split in *Alloteropsis* separates the lineage containing *A. cimicina* from *A. angusta* and *A. semialata* (Fig. 2). These two lineages co‐opted different tissues for the segregation of Rubisco activity and achieved a large proportion of bundle sheath tissue via different modifications (Fig. S1). The evidence therefore strongly supports two independent origins of C_4_ anatomical properties, which is generally accepted as the first step during the C_3_ to C_4_ transition (Fig. 1; Sage et al. 2012; Heckmann et al. 2013). Gene expression analyses show that the two clades use different enzymes for parts of the C_4_ cycle, express different genes encoding the same enzyme family when there is an overlap (Fig. 3), and positive selection analyses show that the enzymes were independently adapted for their C_4_ function (Table 1). We therefore conclude that the different transitions to C_4_ biochemistry occurred independently after the split of these two lineages (Fig. 2). The only exception to the distinctiveness of *A. cimicina* and the two other C_4_ species is the gene *ppc‐1P3_LGT‐M*, used by both *A. cimicina* and some C_4_ *A. semialata* accessions (Fig. 4). This gene is absent from other accessions (Olofsson et al. 2016) and, as such, we previously concluded that it was acquired early during the diversification of the group and then recurrently lost (Christin et al. 2012). This hypothesis is falsified by our dating analyses here, which show that this gene was only recently transferred among species boundaries, likely as a result of a rare hybridization event (Fig. 6).

### ONE INDEPENDENT C_3_ TO C_4_ TRANSITION INCLUDES TWO SEPARATE C_3_+C_4_ TO C_4_ SHIFTS

The C_4_ phenotype is realized in *A. angusta* and *A. semialata* via identical anatomical modifications, using the same enzymes, and the same genes encode these enzymes. Chloroplasts are present in the inner sheaths of all *A. semialata* and *A. angusta* accessions, independent of their photosynthetic type, which suggests that this characteristic represents the ancestral condition for the clade (Fig. 2). The C_4_ cycle is realized using the same set of genes in *A. angusta* and *A. semialata*, which can be explained by convergent evolution (e.g., as indicated for other C_4_ grasses; Christin et al. 2013b) or a single origin of a weak C_4_ cycle (C_3_+C_4_), followed by a reversal to expression levels that resemble the ancestral condition in the C_3_ accessions (Fig. 2). Differentiating these two scenarios would require retracing the origin of the mutations responsible for the increased expression of C_4_ enzymes to identify where they occurred on the phylogeny. Unfortunately, the molecular mechanisms controlling C_4_ gene expression are poorly known, and can involve both cis‐ and transacting elements (Gowik et al. 2004; Brown et al. 2011; Williams et al. 2016).

The positive selection analyses indicate that enzyme adaptation happened independently in *A. angusta* and *A. semialata* (Table 2). Together with the variation observed within the C_4_ *A. semialata* (Fig. 4, S6), this evidence strongly suggests that the biochemical adaptation allowing the transition to a full C_4_ cycle happened recently, and independently in the two species (Fig. 2). The dramatic increase in the proportion of the inner bundle sheath tissue via the proliferation of minor veins is limited to the C_4_ *A. semialata* and *A. angusta* (Fig. S1). The genetic control of these features is unknown, preventing a comparison of the causal mutations. However, the distribution of anatomical characters among grasses indicates that the vast majority of C_4_ lineages that co‐opted the inner bundle sheath increased its proportion via the addition of minor veins (Renvoize 1987; Christin et al. 2013a).

With the current state of knowledge, we hypothesize that the common ancestor of *A. semialata* and *A. angusta* had chloroplasts in the inner bundle sheath, and that this facilitated the emergence of a weak C_4_ cycle via the upregulation of some enzymes. Following their split, *A. angusta* strengthened its C_4_ anatomy via the proliferation of minor veins, and enzyme adaptations led to a strong C_4_ cycle (Fig. 2). In the *A. semialata* lineage, some isolated populations acquired mutations that added minor veins and adapted the enzymes, leading to a C_4_ cycle. Other populations, potentially under pressures linked to the colonization of colder environments (Lundgren et al. 2015), might have lost the weak C_4_ cycle by downregulating the genes (Fig. 2). However, the details of the changes leading to C_3_ photosynthesis in some *A. semialata* will need to be confirmed by comparative genomics, when mutations regulating expression of C_4_ enzymes and anatomy are identified.

### INTROGRESSION OF C_4_ COMPONENTS AMONG SPECIES

Our dating analyses suggest that the gene *pck‐1P1_LGT‐C* that encodes the decarboxylating enzyme PCK was introgressed among some members of *A. semialata* and *A. angusta* (Figs. 2 and 6). The C_4_ cycle carried out before this event was likely based on NADP‐malic enzyme, an enzyme still abundant in the C_3_+C_4_ *A. semialata* and some C_4_ accessions (Fig. 4; Frean et al. 1983). The acquisition of *pck‐1P1_LGT‐C*, a gene already adapted for the C_4_ context, probably added a PCK shuttle, which alters the stoichiometry of the pathway and the spatial distribution of its energy requirements, increasing its efficiency under some conditions (Bellasio and Griffiths 2014; Wang et al. 2014). This important component of the C_4_ cycles of extant *A. semialata* and *A. angusta* populations first evolved its C_4_‐specific properties in the distantly related *Cenchrus* (Fig. S3; Christin et al. 2012), and therefore never evolved within *Alloteropsis*. Instead, it represents the spread of a component of a complex physiology across multiple species boundaries. Therefore, in addition to the possibility that the sequential steps generating a complex physiology can happen on different branches of a species phylogeny (Fig. 2), introgression among close relatives can disconnect the origins of key components from the species tree.

### ON THE INFERENCE OF TRANSITIONS AMONG CHARACTER STATES

Inferences of transitions among character states are a key component of numerous macroevolutionary studies (e.g., Cantalapiedra et al. 2017; Cooney et al. 2017). However, species trees per se are not always able to disentangle the complex scenarios underlying the appearance or losses of multicomponent adaptations, especially when complex phenotypes are modeled as different states of a single character (e.g., Goldberg and Igic 2008; Pardo‐Diaz et al. 2012; Niemiller et al. 2013; Igic and Busch 2013; King and Lee 2015). In the case of photosynthetic transitions within *Alloteropsis* depicted here, considering the photosynthetic type as a binary character would lead to a single C_4_ origin as the most plausible scenario (Ibrahim et al. 2009), and modeling photosynthetic types based on their category of C_4_ cycle does not improve the inference (Washburn et al. 2015). For traits assumed to evolve via sequential stages, the accepted sequence of changes can be incorporated in the model (e.g., Marazzi et al. 2012). However, the power of character modeling remains inherently limited by the small number of informative characters. Decomposing the phenotype into its components can solve this problem, especially when the underlying genetic determinism is considered (Oliver et al. 2012; Niemiller et al. 2013; Glover et al. 2015; Meier et al. 2017), and good mechanistic models exist for the evolution of DNA sequences (Liberles et al. 2013). Violation of model assumptions can still mislead the conclusions, but the multiplication of sources of information, coupled with the possibility to track the history of specific genes independently of the species tree, limits the risks of systematic errors. We therefore suggest that efforts to reconstruct the transitions leading to important traits should integrate as many underlying components as possible. As progresses in genome biology increase data availability and improve our understanding of causal mutations, modeling phenotypes as the results of cumulative changes in genomes will be able to solve the problems raised by the paucity of informative characters.

## Conclusions

In this study, we dissect the genetic and anatomical components of C_4_ photosynthesis in *Alloteropsis*, a genus of grasses with multiple photosynthetic types. Our comparative efforts strongly support at least two independent origins of C_4_ photosynthesis within this genus. The C_4_ phenotype within these separate origins is realized via divergent anatomical modifications, the upregulation of distinct sets of genes, and independent enzyme adaptations. One of these lineages includes a range of photosynthetic types, and based on our analyses, we suggest that some C_4_ components in this group evolved in the shared common ancestor, while others were acquired independently after the lineages diverged. The history of photosynthetic transitions within *Alloteropsis* is furthermore complicated by the introgression of C_4_ genes across species boundaries. This disconnects the spread of C_4_ components from the species tree, and means that the number of origins varies among the different components of the complex C_4_ trait. This scenario is unlikely to have been inferred from traditional macroevolutionary approaches based on species trees alone. We suggest that integrating genomic data and phenotypic details in future studies of character transitions might resolve similarly complicated scenarios in other groups, enabling a better understanding of the trajectories followed during the evolution of novel adaptations.

## Supporting information


**Figure S1**. Comparisons of leaf anatomy in Alloteropsis and relatives.
**Figure S2**. Phylogenetic trees for genes encoding three C4‐related enzymes.
**Figure S3**. Phylogeny of pck‐1P1 genes in Panicoideae.
**Figure S4**. Evolution of ppc‐1P3 genes in Alloteropsis and other Panicoideae.
**Figure S5**. Evolution of ppdk‐1P2 genes in Alloteropsis and other Panicoideae.
**Figure S6**. Evolution of aspat‐3P4 genes in Alloteropsis and other Panicoideae.
**Table S1**. Alloteropsis semialata accessions used in this study.
**Table S2**. Leaf anatomical data for the study species and accessions.
**Table S3**. RNA‐Seq data, NCBI SRA accession numbers, and growth conditions.
**Table S4**. Transcript abundance (in rpkm) for each C4‐related gene and sample.
**Table S5**. Results of positive selection analyses inferring the episodes of enzymatic adaptation in Alloteropsis using only fixed differences.
**Table S6**. Results of positive selection analyses inferring the episodes of enzymatic adaptation in the *A. angusta*/*A. semialata* clade using only fixed differences.
**Methods 1.1**. Plant growth conditions.
**Methods 1.2**. RNA‐Seq protocol.
**Methods 1.3**. Positive selection analysis.
**Methods 1.4**. Alignment and filtering

Supplementary Material
