# Supplementary material for: Introgression and repeated co‐option facilitated the recurrent emergence of C4 photosynthesis among close relatives
Source: Evolution. 2017 Apr 28;71(6):1541–55. doi: 10.1111/evo.13250 (PMC5488178; doi:10.1111/evo.13250)
Supplement: Supplementary file 1 — Figure S1. Comparisons of leaf anatomy in Alloteropsis and relatives. Figure S2. Phylogenetic trees for genes encoding three C4‐related enzymes. Figure S3. Phylogeny of pck‐1P1 genes in Panicoideae. Figure S4. Evolution of ppc‐1P3 genes in Alloteropsis and other Panicoideae. Figure S5. Evolution of ppdk‐1P2 genes in Alloteropsis and other Panicoideae. Figure S6. Evolution of aspat‐3P4 genes in Alloteropsis and other Panicoideae. Table S1. Alloteropsis semialata accessions used in this study. Table S2. Leaf anatomical data for the study species and accessions. Table S3. RNA‐Seq data, NCBI SRA accession numbers, and growth conditions. Table S4. Transcript abundance (in rpkm) for each C4‐related gene and sample. Table S5. Results of positive selection analyses inferring the episodes of enzymatic adaptation in Alloteropsis using only fixed differences. Table S6. Results of positive selection analyses inferring the episodes of enzymatic adaptation in the A. angusta/A. semialata clade using only fixed differences. Methods 1.1. Plant growth conditions. Methods 1.2. RNA‐Seq protocol. Methods 1.3. Positive selection analysis. Methods 1.4. Alignment and filtering [file EVO-71-1541-s001.pdf]

Supplementary Material for:

Introgression and repeated co-option facilitated the recurrent emergence of C_4 photosynthesis among close relatives

Authors: Luke T. Dunning, Marjorie R. Lundgren, Jose J Moreno-Villena, Mary Namaganda, Erika J. Edwards, Patrik Nosil, Colin P. Osborne, Pascal-Antoine Christin

This supplementary material contains supplementary methods, six figures and four tables:

Figure S1: Comparisons of leaf anatomy in *Alloteropsis* and relatives.

Figure S2: Phylogenetic trees for genes encoding three C_4-related enzymes.

Figure S3: Phylogeny of *pck-1P1* genes in Panicoideae.

Figure S4: Evolution of *ppc-1P3* genes in *Alloteropsis* and other Panicoideae.

Figure S5: Evolution of *ppdk-1P2* genes in *Alloteropsis* and other Panicoideae.

Figure S6: Evolution of *aspat-3P4* genes in *Alloteropsis* and other Panicoideae.

Table S1: *Alloteropsis semialata* accessions used in this study.

Table S2: Leaf anatomical data for the study species and accessions.

Table S3: RNA-Seq data, NCBI SRA accession numbers, and growth conditions.

Table S4: Transcript abundance (in rpkm) for each C_4-related gene and sample.

Table S5: Results of positive selection analyses inferring the episodes of enzymatic adaptation in *Alloteropsis* using only fixed differences.

Table S6: Results of positive selection analyses inferring the episodes of enzymatic adaptation in the *A. angusta/A. semialata* clade using only fixed differences.

Table S7: Effect of gene tree topology on the positive selection analyses in *Alloteropsis*.

Table S8:Effect of gene tree topology on the positive selection analyses in the *A. angusta/A. semialata* clade.

Supplementary Methods

1.1 Plant growth conditions

Alloteropsis semialata, A. angusta, and *Panicum pygmaeum* plants were grown from seeds or propagated vegetatively from cuttings collected in the field. All individuals were maintained in controlled environment chambers (Conviron BDR16; Manitoba, Canada) set to 60% relative humidity, 500 µmol m^{-2} s^{-1} photosynthetic photon flux density (PPFD), and 25/20°C day/night temperatures with 14h of light at the University of Sheffield. Plants were grown in John Innes No. 2 potting compost (John Innes Manufacturers Association, Reading, England), maintained under well-watered conditions, and fertilised every two weeks (Scotts Evergreen Lawn Food; The Scotts Company, Surrey, England). After a minimum of 30 days in the above conditions, samples were taken for RNA-Seq and leaf anatomy. For RNA-Seq, certain individuals were then were resampled after 30 days under a 10-hour photoperiod (Table S3). Leaf samples from *A. cimicina* were collected from two individual plants grown under ambient glasshouse conditions at Brown University.

1.2 RNA-Seq protocol

Total RNA was extracted from *A. semialata, A. angusta,* and *P. pygmaeum* samples using the RNeasy Plant Mini Kit (Qiagen, Hilden, Germany), following the manufacturer's protocol. An on-column DNA digestion step was performed using the RNase-Free Dnase Set (Qiagen, Hilden, Germany). Total RNA was eluted in RNAse-free water with 20 U/µL of SUPERase-IN RNase Inhibitor (Life Technologies, Carlsbad, CA). RNA quality and concentration were determined using the RNA 6000 Nano kit with an Agilent Bioanalyzer 2100 (Agilent Technologies, Palo Alto, California). Extractions used for library preparation contained at least 0.5 µg of total RNA, with an RNA integrity number (RIN) greater than 6.5. Each sample was prepared individually using the TruSeq RNA Library Preparation Kit v2 (Illumina, San Diego, CA), following the manufacturer's protocol with an eight-minute fragmentation step. Indexed libraries were paired-end sequenced by

the Sheffield Diagnostic Genetics Service on an Illumina HiSeq 2500 platform for 100 cycles in rapid mode, with 24 libraries pooled per lane of the flow cell. The two *A. cimicina* leaf samples were sequenced as described in Christin et al. (2015).

The RNA-seq data were filtered and assembled using the Agalma pipeline v.0.5.0 with default parameters (Dunn et al. 2013). In brief, this pipeline removes the reads that are low quality (Q <30), adaptor contaminated, or correspond to rRNA, prior to constructing *de novo* assemblies using Trinity (version trinityrnaseq_r20140413p1; Grabherr et al. 2011). One assembly was generated per genotype, using all reads available for each accession (Table S3). All raw RNA-Seq data have been deposited in the NCBI Sequence Read Archive (project identifier SRP072730, Table S3), and transcriptome assemblies are deposited in the NCBI Transcriptome Shotgun Assembly repository (Bioproject PRJNA310121). To generate transcript abundances, the paired-end reads from each library were mapped back onto their respective reference transcriptome assembly using bowtie2 v.2.0.5 (Langmead and Salzberg 2012).

In total, 38 individually sequenced RNA-Seq libraries from 14 different accessions/species generated over 300 million 100 bp paired-end reads. This represents 66.44 Gb of data, with a mean of 1.75 Gb per library (SD = 1.56 Gb; Table S3). Over 80% of the data were kept after removing low-quality reads and ribosomal RNA sequences (Table S3). One transcriptome was assembled per genotype, pooling all the reads obtained for each genotype (mean per genotype = 3.87 Gb, SD = 1.76 Gb). The 14 assembled transcriptomes were all of comparable quality, with a mean of 44,578 trinity 'unigenes' (i.e. putative loci in the transcriptome assembly; SD = 6,905), 65,725 contigs (SD = 12,282), and a 1,543 bp N50 (SD = 167 bp).

1.3 Positive selection analysis

For each gene lineage, additional sequences were retrieved from complete published genomes for Panicoideae, the NCBI non-redundant nucleotide database, and other published transcriptomes (Bräutigam et al. 2014). The *pck-1P1* gene is expressed at extremely low levels in the C_4 *A.*

semialata and *A. angusta* (see Results), and was therefore not assembled as part of the transcriptomes. As an alternative, we used coding sequences previously generated by Sanger sequencing when available (Christin et al. 2012), or manually assembled PCK coding regions from low coverage genome sequencing data (Olofsson et al. 2016). Each set of genes was aligned as codons using ClustalW (Thompson et al. 2002), and the resulting alignments were manually refined, including truncating the 5' or 3' ends to remove poorly aligned segments. A phylogenetic tree was inferred on 3rd positions of codons, using PhyML, with the GTR+G+I model and 100 bootstrap pseudoreplicates. The gene tree topologies were used for subsequent selection analyses, after removing sequences belonging to C_4 species other than *Alloteropsis* to avoid an influence of positive selection in these taxa affecting our conclusion. C_3 species outside *Alloteropsis* were however kept for positive selection analyses.

For genes not involved in the C_4 cycle of *A. cimicina,* we repeated the positive selection analyses to distinguish between a single (common ancestor of *A. angusta* and *A. semialata*) and two episodes of adaptive evolution (*A. angusta* and C_4 *A. semialata* separately) within the *A. angusta*/*A.semialata* clade. This was also preformed with the hypothesis of positive selection acting only in *A. angusta*. In addition, we also performed these tests on the genes for which selection was detected on the branch leading to *A. cimicina*, after excluding *A. cimicina* sequences, to evaluate the possibility that selection operated on different sites in the different lineages.

1.4 Alignment and filtering

Stringent alignment and filtering methods were used to ensure reliable alignments of each gene family for phylogenetic inference. First, sequences within each gene family were translated and aligned as proteins with four different assemblers (maaft, muscle, kalign, t-coffee) using m-coffee (Wallace et al. 2006) as part of the t-coffee package v.11.0 (Notredame et al. 2000). Consensus alignments from the four different methods were then trimmed so that only amino acids aligned in

the same position by all of the assemblers were retained. Alignments were further parsed using the tcs residue filter (Chang et al. 2014), only retaining the highest confidence residues. The trimmed protein alignments were reverse-translated into nucleotide alignments using the original sequences, and further filtered using gblocks v.0.91 (parameters: -t=c -b2=b1 -b5=h; Castresana 2000). Finally, sequences shorter than 100 bp were removed, and maximum likelihood trees were inferred with PhyML. Putative groups of Panicoideae co-orthologs were identified as monophyletic groups that contained only sequences from Panicoideae species. The alignment process was repeated for each of these groups of putative co-orthologs, starting again from the initial untrimmed sequences, producing high quality alignments for each individual group. Subsequent analyses were restricted to groups containing at least one sequence of each *Alloteropsis* species and *Sorghum* (used as the outgroup), and phylogenetic trees were again inferred with PhyML. Datasets where at least one of the six Panicoideae species (*Sorghum*, *Setaria*, *P. pygmaeum*, *A. cimicina*, *A. angusta*, and *A. semialata*), the *Alloteropsis* genus, or the *semialata*/*angusta* clade was not monophyletic in the maximum likelihood tree were discarded to remove genes that were duplicated after the divergence from the outgroup or poorly informative datasets. Of the 4,969 datasets originally screened, 1,042 were discarded because at least one of *Sorghum*, *Setaria*, *P. pygmaeum*, or the *Alloteropsis* genus was not monophyletic. These include potential cases of paralogy problems, sequencing or assembly errors, and poor phylogenetic resolution in the deep nodes of the trees. A further 1,130 datasets were discarded because one of the *Alloteropsis* species or the *A. semialata*/*A. angusta* clade was not monophyletic. This category includes potential *Alloteropsis*-specific duplicates and datasets lacking resolution among these closely-related taxa. While it cannot be excluded that some of these incongruences reflect true biological phenomena, the remaining 2,797 datasets (56% of the original ones) represent reliable markers for dating analyses. Finally, we removed species-specific duplicates, or transcript variants, by only retaining the longest sequence for each accession when several sequences from that accession formed a monophyletic clade.

Captions for Supplementary Figures

Figure S1: Comparisons of leaf anatomy in *Alloteropsis* and relatives.

Leaf cross-sections are shown for each group. The red bar at the bottom represents 0.5 mm. Black arrows indicate mesophyll cells (M), red arrows inner sheath (IS) cells and orange arrows outer sheath (OS) cells. The species and photosynthetic type are indicated on the right.

Figure S2: Phylogenetic trees for genes encoding three C_4-related enzymes.

These maximum likelihood trees show the relationships among genes used to circumscribe grass co-orthologs for the phylogenetic annotation of contigs. The trees are shown for three families changed compared to Christin et al. (2013, 2015); **A)** NAD-malate dehydrogenase (*nadmdh-4*), **B)** phosphoenolpyruvate-phosphate translocator (*ppt*)/triosephosphate-phosphate translocator (*tpt*)/glucose-6-phosphate/phosphate translocator (*gpt*), **C)** Sodium bile acid symporter (*sbas*). For each tree, grass co-orthologs are delimited on the right, with names following the approach of Christin et al. (2015). Bootstrap values are indicated near branches

Figure S3: Phylogeny of *pck-1P1* genes in Panicoideae.

This phylogenetic tree was inferred on 3rd positions of codons. Bootstrap values are indicated near branches. Branches leading to genes that have been co-opted for C_4 photosynthesis are in green, following Christin et al. (2012). Tribes are delimited on the right. The laterally acquired *pck-1P1-C* gene is indicated.

Figure S4: Evolution of *ppc-1P3* genes in *Alloteropsis* and other Panicoideae.

This phylogenetic tree was inferred on 3rd positions of codons of *ppc-1P3* genes of Panicoideae. Bootstrap values are indicated near branches. Names of C_4 accessions are in bold. Gray branches were pruned before selection tests. Positive selection was detected on the thick branch. Groups of *Alloteropsis* genes are delimited on the right.

Figure S5: Evolution of *ppdk-1P2* genes in *Alloteropsis* and other Panicoideae.

This phylogenetic tree was inferred on 3rd positions of codons of *ppdk-1P2* genes of Panicoideae. Bootstrap values are indicated near branches. Names of C_4 accessions are in bold. Gray branches were pruned before selection tests. Positive selection was detected on the thick branch. Amino acid positions with a posterior probability >0.90 of being under positive selection are indicated on the right, asterisks indicate positions with a posterior probability >0.95, with those associated with C_4 accessions in gray. Positions are indicated on the top, based on *Sorghum* gene Sb09g019930.1.

Figure S6: Evolution of *aspat-3P4* genes in *Alloteropsis* and other Panicoideae.

This phylogenetic tree was inferred on 3rd positions of codons of *aspat-3P4* genes of Panicoideae. Bootstrap values are indicated near branches. Names of C_4 accessions are in bold. Gray branches were pruned before selection tests. Positive selection was detected on thick branches. Amino acid positions with a posterior probability >0.90 of being under positive selection are indicated on the right, asterisks indicate positions with a posterior probability >0.95, with those associated with C_4 accessions in gray. Positions are indicated on the top, based on *Sorghum* gene Sb03g035220.1. Asterisks indicate positions with a posterior probability >0.9.

Table S1: *Alloteropsis semialata* accessions used in this study[1].

ID	Sample name	Country	Latitude	Longitude	Type	$\delta^{13}C$
RSA5	KWT3	South Africa	-32.70	27.53	C_3	-26.3
TAN2	L01	Tanzania	-5.63	32.69	C_3+C_4	-26.3
TAN1	L04	Tanzania	-8.51	35.17	C_3+C_4	-23.1
TAN4	L02	Tanzania	-9.04	32.48	C_4	-11.4
BUR1	BF3	Burkina Faso	10.85	-4.83	C_4	-11.3
MAD1	Maj	Madagascar	-15.67	46.37	C_4	-11.8
RSA3	MDB8	South Africa	-25.76	29.47	C_4	-12.7
RSA4	SFD3	South Africa	-28.39	29.04	C_4	-12.7
AUS1	Aus2	Australia	-19.62	146.96	C_4	-12.1
TPE1	TW10	Taiwan	24.47	120.72	C_4 [2]	-11.8

[1] Collection localities and photosynthetic type with the diagnostic physiology data come from Lundgren et al. (2016). Stable isotope data from Lundgren et al. (2015). [2]Inferred from stable isotope data, adjusted for anthropogenic CO_2 sources per Lundgren et al. (2016).

Table S2: Leaf anatomical data for the study species and accessions[1].

Species/Accession	Pathway	C_4 sheath	minor veins[2]	IVD (µm)	nb.M	OS.width (µm)	IS.width (µm)	OS:IS
Entolasia marginata[3]	C_3	na	absent	255.5	7.5	24.9	4.7	5.3
Panicum pygmaeum[3]	C_3	na	absent	219.9	7.0	27.9	6.2	4.5
Alloteropsis semialata, RSA5	C_3	na	absent	186.2	7.4	12.2	6.8	1.8
Alloteropsis semialata, TAN2	C_3+C_4	inner	absent	167.4	4.4	12.4	10.1	1.2
Alloteropsis semialata, TAN1	C_3+C_4	inner	absent	159.8	5.4	11.8	9.4	1.3
Alloteropsis semialata, TAN4	C_4	inner	present	127.8	2.7	9.7	14.0	0.7
Alloteropsis semialata, AUS1	C_4	inner	present	79.1	1.8	10.4	12.0	0.9
Alloteropsis semialata, BUR1	C_4	inner	present	77.9	1.3	11.3	10.6	1.1
Alloteropsis semialata, MAD1	C_4	inner	present	92.9	1.8	9.7	13.3	0.7
Alloteropsis semialata, RSA3	C_4	inner	present	86.0	1.8	9.6	11.7	0.8
Alloteropsis semialata, RSA4	C_4	inner	present	97.2	1.8	8.4	13.3	0.6
Alloteropsis semialata, TPE1	C_4	inner	present	84.5	1.2	8.7	7.2	1.2
Alloteropsis angusta	C_4	inner	present	83.4	1.0	9.5	9.8	1
Alloteropsis cimicina[3]	C_4	outer	absent	292.3	3.4	46.7	6.0	7.8
Alloteropsis paniculata[3]	C_4	outer	absent	198.0	2.7	43.9	5.6	7.8

[1] Column headings and abbreviations: C_4 sheath, bundle sheath used for CO_2 reduction; IVD, interveinal distance; nb.M, number of mediolateral mesophyll cells separating vein units; OS.width, the width of the outer bundle sheath cells; IS.width, the width of the inner bundle sheath cells; OS:IS is the ratio of outer to inner bundle sheath cell size. [2] Minor veins are considered 4th and 5th order veins here, while the midrib, secondary and tertiary vein orders are excluded from this category. [3] Data taken from Christin et al. 2013.

Table S3: RNA-Seq data, NCBI SRA accession numbers, and growth conditions.

Genotype	Species	SRA accession	Tissue	Photoperiod	Raw PE Reads	Clean PE Reads	No. Trinity contigs
ACIM	*A. cimicina*	SRR3994072	Leaf	Glasshouse	36087907	27351333	51195
		SRR3994073	Leaf	Glasshouse	28714973	4854902	
ANG33	*A. angusta*	SRR3994075	Leaf	14hr	7334658	6612013	72468
ANG48	*A. angusta*	SRR3994077	Leaf	14hr	7498597	6826154	71835
AUS1	*A. semialata*	SRR3321311	Leaf	10hr	5308967	4675722	54197
		SRR3322358	Leaf	14hr	11184717	9624467	
		SRR3322714	Root	14hr	3950267	3613034	
BUR1	*A. semialata*	SRR3322990	Leaf	10hr	11153698	9575675	75444
		SRR3322973	Leaf	14hr	16859344	14948845	
		SRR3323003	Root	14hr	2223260	3042111	
RSA5	*A. semialata*	SRR3323066	Leaf	10hr	5500526	2402701	63273
		SRR3323049	Leaf	14hr	13458893	12135442	
		SRR3323067	Root	14hr	3107385	2915544	
TAN2	*A. semialata*	SRR3323068	Leaf	10hr	17997033	16131010	74639
		SRR3323088	Leaf	14hr	5423680	4804035	
		SRR3323114	Root	14hr	4282037	4018356	
TAN4	*A. semialata*	SRR3323124	Leaf	14hr	3218981	3218981	58125
		SRR3323125	Root	14hr	4689624	4689624	
TAN1	*A. semialata*	SRR3323127	Leaf	10hr	25015574	22153077	74400
		SRR3323128	Root	10hr	11154350	9983220	
		SRR3323129	Root	14hr	3137368	2928130	
MAD1	*A. semialata*	SRR3323131	Leaf	10hr	1338699	1146407	75444
		SRR3323132	Leaf	14hr	2546427	2190484	
		SRR3323133	Root	14hr	10980770	9826198	
		SRR3323134	Root	10hr	3460229	3201082	
RSA3	*A. semialata*	SRR3323186	Leaf	10hr	5001282	2021239	74023
		SRR3323137	Leaf	14hr	11922494	10671710	
		SRR3323187	Root	14hr	3950750	4185063	
PPYG	*P. pygmaeum*	SRR3330791	Leaf	14hr	4093890	3793221	72117
		SRR3323220	Leaf	14hr	8925603	8087404	
		SRR3330803	Root	14hr	4106624	3482683	
		SRR3330803	Root	14hr	2026469	1752009	
RSA4	*A. semialata*	SRR3323240	Leaf	10hr	4467544	3470414	87362
		SRR3323220	Leaf	14hr	16357704	14849292	
		SRR3323241	Root	14hr	3248828	4614268	
TPE1	*A. semialata*	SRR3323242	Leaf	10hr	12422286	6546514	57350
		SRR3323243	Leaf	14hr	7457117	10995742	
		SRR3323244	Root	14hr	2604885	3699333	

Table S5: Results of positive selection analyses inferring the episodes of enzymatic adaptation in *Alloteropsis*[1] using only codons with fixed nucleotides for each photosynthetic type within *A. semialata* and *A. angusta.*

				One origin		Two origins		Three origins		Only *A. cimicina*	
**Gene**	**Number of sequences**	**Number codons removed**	**Site model M1a**	**BSA**	**BSA1**	**BSA**	**BSA1**	**BSA**	**BSA1**	**BSA**	**BSA1**
aspat-2P3	7	23	**0.00***	4.05	4.05	4.05	4.05	3.54	3.54	4.05	4.05
nadpme-1P4	8	29	25.14	6.13	4.43	6.13	4.43	11.68	9.83	3.77	**0.00***
ppdk-1P2	8	48	26.34	11.49	8.91	11.49	8.91	9.67	4.73	4.27	**0.00***
alaat-1P5	7	33	**0.00***	4.03	4.03	4.04	4.04	3.61	3.61	4.04	4.04

[1] The ΔAICc values compared to the best fit model for that gene are shown. The most appropriate model is indicated with an asterisk, with the null model (M1a) only rejected if the ΔAICc was at least 5.22 (equivalent to a p-value of 0.01 with a likelihood ratio test with df = 2). Two branch-site models were used to test for a relaxation of purifying selection (BSA), and potential positive selection (BSA1).

Table S6: Results of positive selection analyses inferring the episodes of enzymatic adaptation in the *A. angusta/A. semialata* clade[1] using only codons with fixed nucleotides for each photosynthetic type within *A. semialata* and *A. angusta.*

Gene	Number of sequences	Number codons removed	Site model M1a	One origin		Two origins		Only *A. angusta*	
				**BSA**	**BSA1**	**BSA**	**BSA1**	**BSA**	**BSA1**
aspat-3P4	7	13	8.62	12.67	12.67	**0.00***	**0.00***	**0.00***	**0.00***
nadpme-1P4	7	29	**0.00***	4.04	4.04	3.47	1.19	3.71	3.71
ppc-1P3	6	70	65.71	23.63	22.33	9.50	5.83	6.17	**0.00***
ppdk-1P2	7	48	**0.00***	4.02	4.02	3.38	3.38	3.20	3.20

[1] The ΔAICc values compared to the best fit model for that gene are shown. The most appropriate model is indicated with an asterisk, with the null model (M1a) only rejected if the ΔAICc 5.22 (equivalent to a p-value of 0.01 with a likelihood ratio test with df = 2). Two branch-site models were used to test for a relaxation of purifying selection (BSA), and potential positive selection (BSA1).

Table S7: Effect of gene tree topology on the conclusions of the positive selection analyses in *Alloteropsis*[1].

Gene	Site model M1a	One origin		Two origins		Three origins		Only *A. cimicina*	
		BSA	BSA1	BSA	BSA1	BSA	BSA1	BSA	BSA1
aspat-2P3	**100**	0	0	0	0	0	0	0	0
nadpme-1P4	0	0	0	0	0	0	0	0	**100**
ppdk-1P2	0	0	0	0	0	0	0	0	**100**
alaat-1P5	**100**	0	0	0	0	0	0	0	0

[1] The number of topologies favouring each modelled, out of 100 bootstrap pseudoreplicates, is indicated.

Table S8: Assessing the effect of gene tree topology on the conclusions of the positive selection analyses within *A. semialata* and *A. angusta*[1].

Gene	Site model M1a	One origin BSA	One origin BSA1	Two origins BSA	Two origins BSA1	Only *A. cimicina* BSA	Only *A. cimicina* BSA1
aspat-3P4	0	0	0	0	**100**	0	0
nadpme-1P4	**2**	0	0	0	**98**	0	0
ppc-1P3	0	0	0	0	0	0	**100**
ppdk-1P2	**100**	0	0	0	0	0	0

[1] The number of topologies favouring each modelled, out of 100 bootstrap pseudoreplicates, is indicated.

Supplementary references:

Bräutigam, A., S. Schliesky, C. Külahoglu, C. P. Osborne, and A. P. Weber. 2014. Towards an integrative model of C_4 photosynthetic subtypes: insights from comparative transcriptome analysis of NAD-ME, NADP-ME, and PEP-CK C_4 species. *J Exp Bot* **65**:3579-3593.

Castresana, J. 2000. Selection of conserved blocks from multiple alignments for their use in phylogenetic analysis. *Mol Biol Evol* **17**:540–552.

Chang, J.M., Di Tommaso, P., Notredame, C. 2014. TCS: A new multiple sequence alignment reliability measure to estimate alignment accuracy and improve phylogenetic tree reconstruction. *Mol Biol Evol* **31**:1625-1637.

Christin, P. A., E. J. Edwards, G. Besnard, S. F. Boxall, R. Gregory, E. A. Kellogg, J. Hartwell, and C. P. Osborne. 2012. Adaptive evolution of C_4 photosynthesis through recurrent lateral gene transfer. *Curr Biol* **22**:445-449.

Christin, P. A., S. F. Boxall, R. Gregory, E. J. Edwards, J. Hartwell, and C. P. Osborne. 2013. Parallel recruitment of multiple genes into C_4 photosynthesis. *Genome Biol Evol* **5**:2174-2187.

Christin, P. A., M. Arakaki, C. P. Osborne, and E. J. Edwards. 2015. Genetic enablers underlying the clustered evolutionary origins of C_4 photosynthesis in angiosperms. *Mol Biol Evol* **32**:846-858.

Dunn, C. W., M. Howison, and F. Zapata. 2013. Agalma: an automated phylogenomics workflow. *BMC Bioinformatics* **14**:330.

Grabherr, M. G., B. J. Haas, M. Yassour, J. Z. Levin, D. A. Thompson, I. Amit, X. Adiconis, L. Fan, R. Raychowdhury, Q. Zeng et al. 2011. Full-length transcriptome assembly from RNA-seq data without a reference genome. *Nature Biotechnol* **29**:644-652.

Langmead, B., and S. Salzberg. 2012. Fast gapped-read alignment with Bowtie 2. *Nat Methods* **9**:357-359.

Lundgren, M. R., G. Besnard, B. S. Ripley, C. E. R. Lehmann, D. S. Chatelet, R. G. Kynast, M. Namaganda, M. S. Vorontsova, R. C. Hall, J. Elia, et al. 2015. Photosynthetic innovation broadens the niche within a single species. *Ecol Lett* **18**:1021-1029.

Lundgren, M. R., P. A. Christin, E. Gonzalez Escobar, B. S. Ripley, G. Besnard, C. M. Long, P. W. Hattersley, R. P. Ellis, R. C. Leegood, and C. P. Osborne CP. 2016. Evolutionary implications of C_3-C_4 intermediates in the grass *Alloteropsis semialata*. *Plant Cell Environ* **39**:1874-85

Notredame, C., Higgins, D.G., Heringa, J. 2000. T-Coffee: A novel method for fast and accurate multiple sequence alignment. *J Mol Biol* **302**:205–17.

Olofsson, J. K., M. Bianconi, G. Besnard, L. T. Dunning, M. R. Lundgren, H. Holota, M. S. Vorontsova, O. Hidalgo, I. J. Leitch, P. Nosil, C. P. Osborne, and P. A. Christin. 2016. y reveals the intraspecific spread of adaptive mutations for a complex traitptive mutations for a

complex trait. *Mol Ecol* **24**: 6107-6123

Thompson, J. D., D. G. Higgins, and T. J. Gibson. 1994. CLUSTAL W: improving the sensitivity of progressive multiple sequence alignment through sequence weighting, position-specific gap penalties and weight matrix choice. *Nucleic Acids Res* **22**:4673-4680.

Wallace, I.M., O'Sullivan, O., Higgins, D.G., Notredame, C. 2006. M-Coffee: combining multiple sequence alignment methods with T-Coffee. *Nucleic Acids Res* **34**:1692–9.

Fig. S1

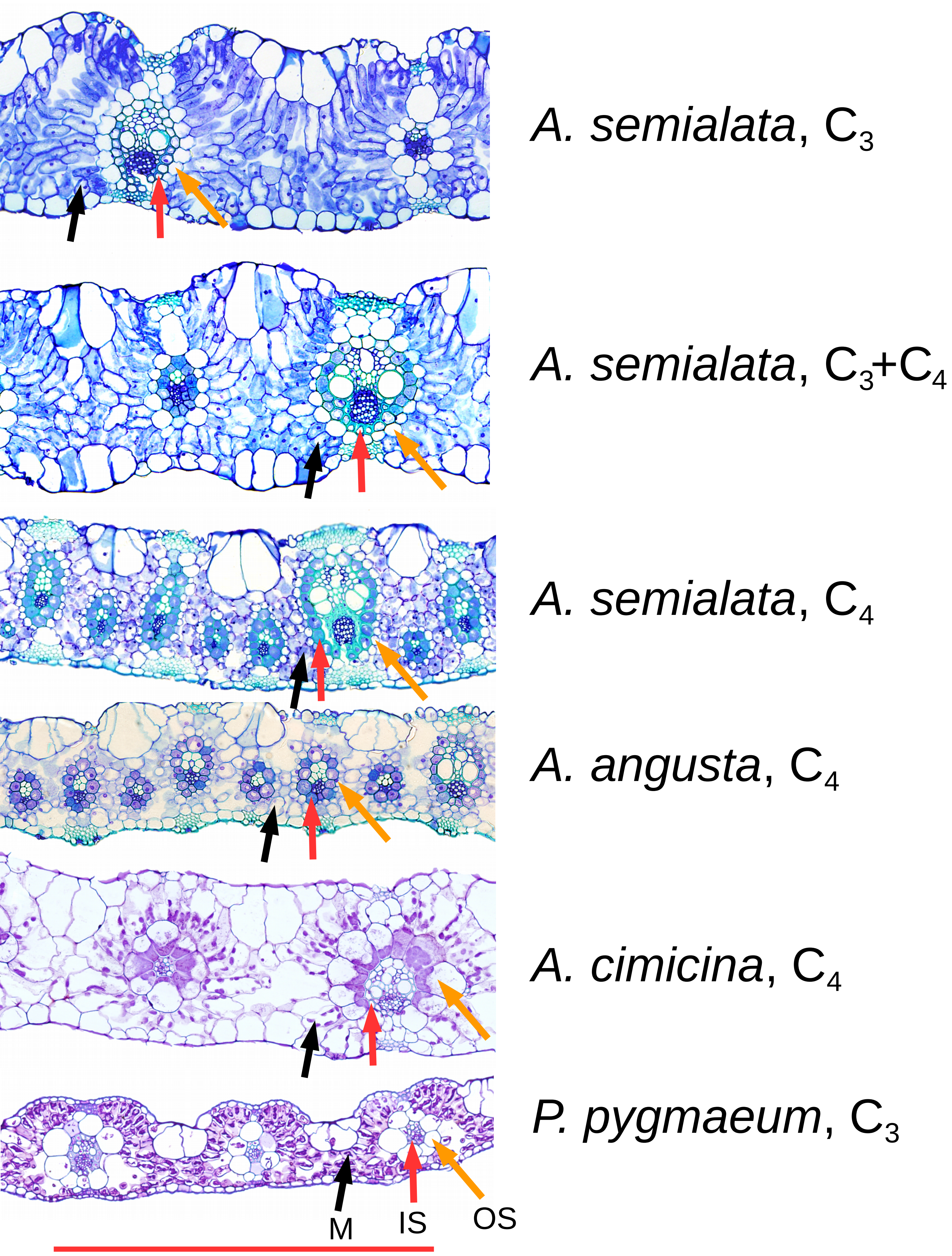
A. semialata, C3
A. semialata, C3+C4
A. semialata, C4
A. angusta, C4
A. cimicina, C4
P. pygmaeum, C3
M
IS
OS

Fig. S2 A

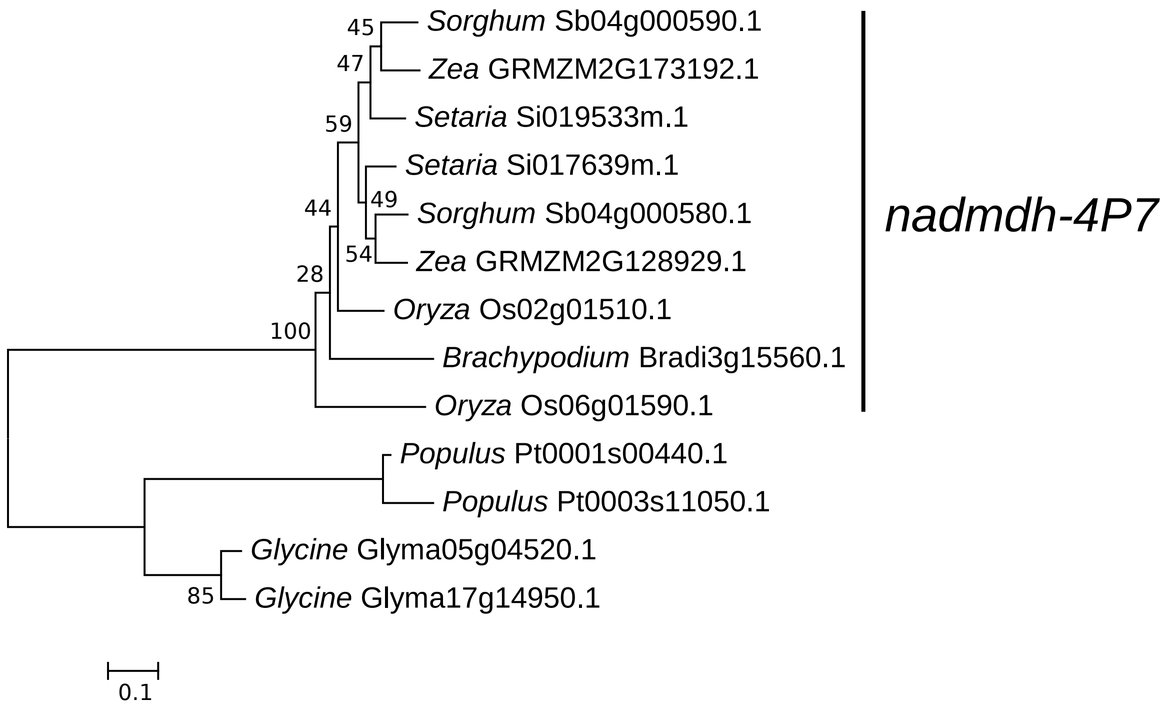
Sorghum Sb04g000590.1
Zea GRMZM2G173192.1
Setaria Si019533m.1
Setaria Si017639m.1
Sorghum Sb04g000580.1
Zea GRMZM2G128929.1
Oryza Os02g01510.1
Brachypodium Bradi3g15560.1
Oryza Os06g01590.1
Populus Pt0001s00440.1
Populus Pt0003s11050.1
Glycine Glyma05g04520.1
Glycine Glyma17g14950.1
45
47
59
49
44
54
28
100
85
0.1
nadmdh-4P7

Fig. S2 B

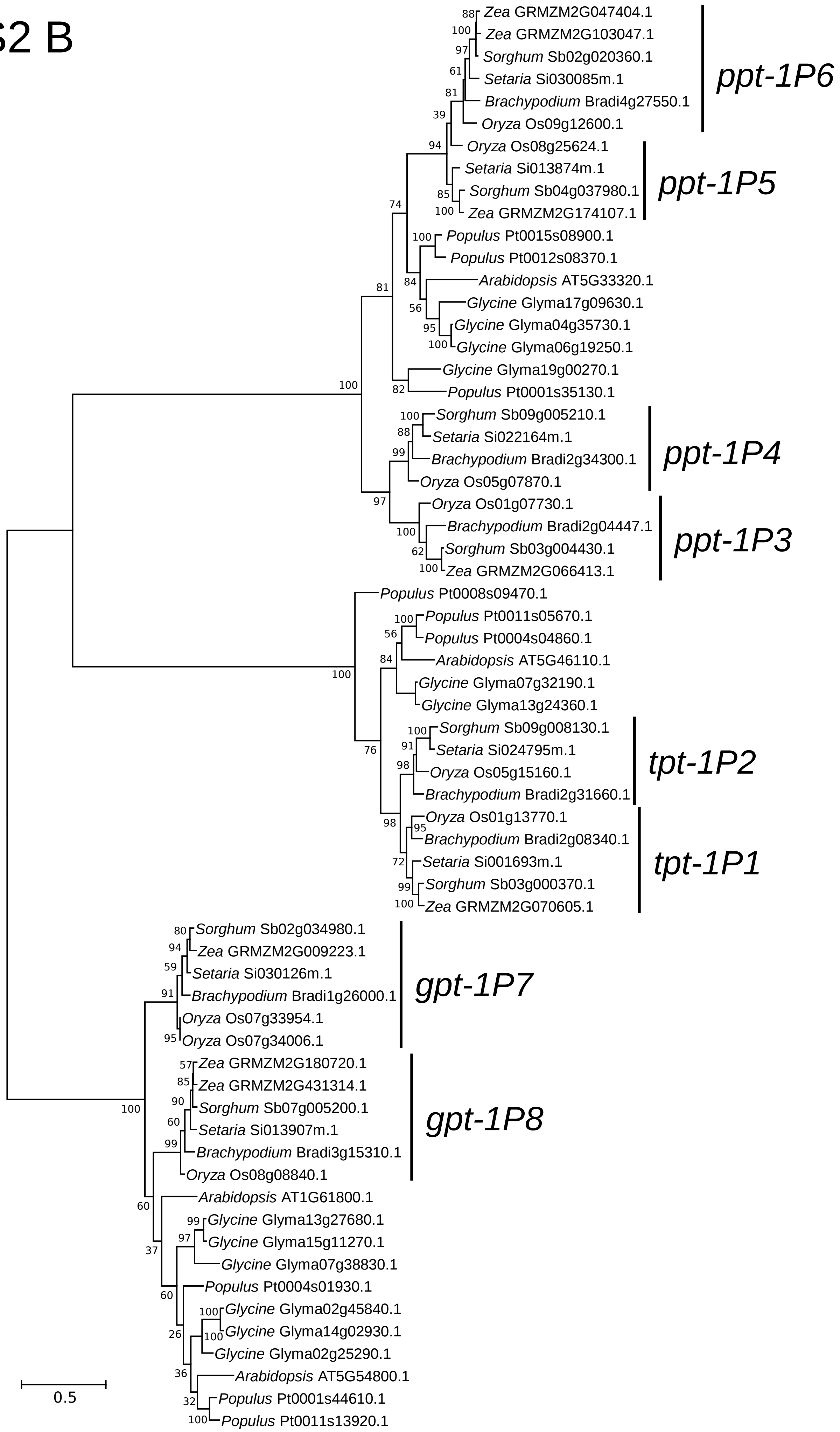

Zea GRMZM2G047404.1
Zea GRMZM2G103047.1
Sorghum Sb02g020360.1
Setaria Si030085m.1
Brachypodium Bradi4g27550.1
Oryza Os09g12600.1
ppt-1P6
Oryza Os08g25624.1
Setaria Si013874m.1
Sorghum Sb04g037980.1
Zea GRMZM2G174107.1
ppt-1P5
Populus Pt0015s08900.1
Populus Pt0012s08370.1
Arabidopsis AT5G33320.1
Glycine Glyma17g09630.1
Glycine Glyma04g35730.1
Glycine Glyma06g19250.1
Glycine Glyma19g00270.1
Populus Pt0001s35130.1
Sorghum Sb09g005210.1
Setaria Si022164m.1
Brachypodium Bradi2g34300.1
Oryza Os05g07870.1
ppt-1P4
Oryza Os01g07730.1
Brachypodium Bradi2g04447.1
Sorghum Sb03g004430.1
Zea GRMZM2G066413.1
ppt-1P3
Populus Pt0008s09470.1
Populus Pt0011s05670.1
Populus Pt0004s04860.1
Arabidopsis AT5G46110.1
Glycine Glyma07g32190.1
Glycine Glyma13g24360.1
Sorghum Sb09g008130.1
Setaria Si024795m.1
Oryza Os05g15160.1
Brachypodium Bradi2g31660.1
tpt-1P2
Oryza Os01g13770.1
Brachypodium Bradi2g08340.1
Setaria Si001693m.1
Sorghum Sb03g000370.1
Zea GRMZM2G070605.1
tpt-1P1
Sorghum Sb02g034980.1
Zea GRMZM2G009223.1
Setaria Si030126m.1
Brachypodium Bradi1g26000.1
Oryza Os07g33954.1
Oryza Os07g34006.1
gpt-1P7
Zea GRMZM2G180720.1
Zea GRMZM2G431314.1
Sorghum Sb07g005200.1
Setaria Si013907m.1
Brachypodium Bradi3g15310.1
Oryza Os08g08840.1
gpt-1P8
Arabidopsis AT1G61800.1
Glycine Glyma13g27680.1
Glycine Glyma15g11270.1
Glycine Glyma07g38830.1
Populus Pt0004s01930.1
Glycine Glyma02g45840.1
Glycine Glyma14g02930.1
Glycine Glyma02g25290.1
Arabidopsis AT5G54800.1
Populus Pt0001s44610.1
Populus Pt0011s13920.1
0.5

Fig. S2 C

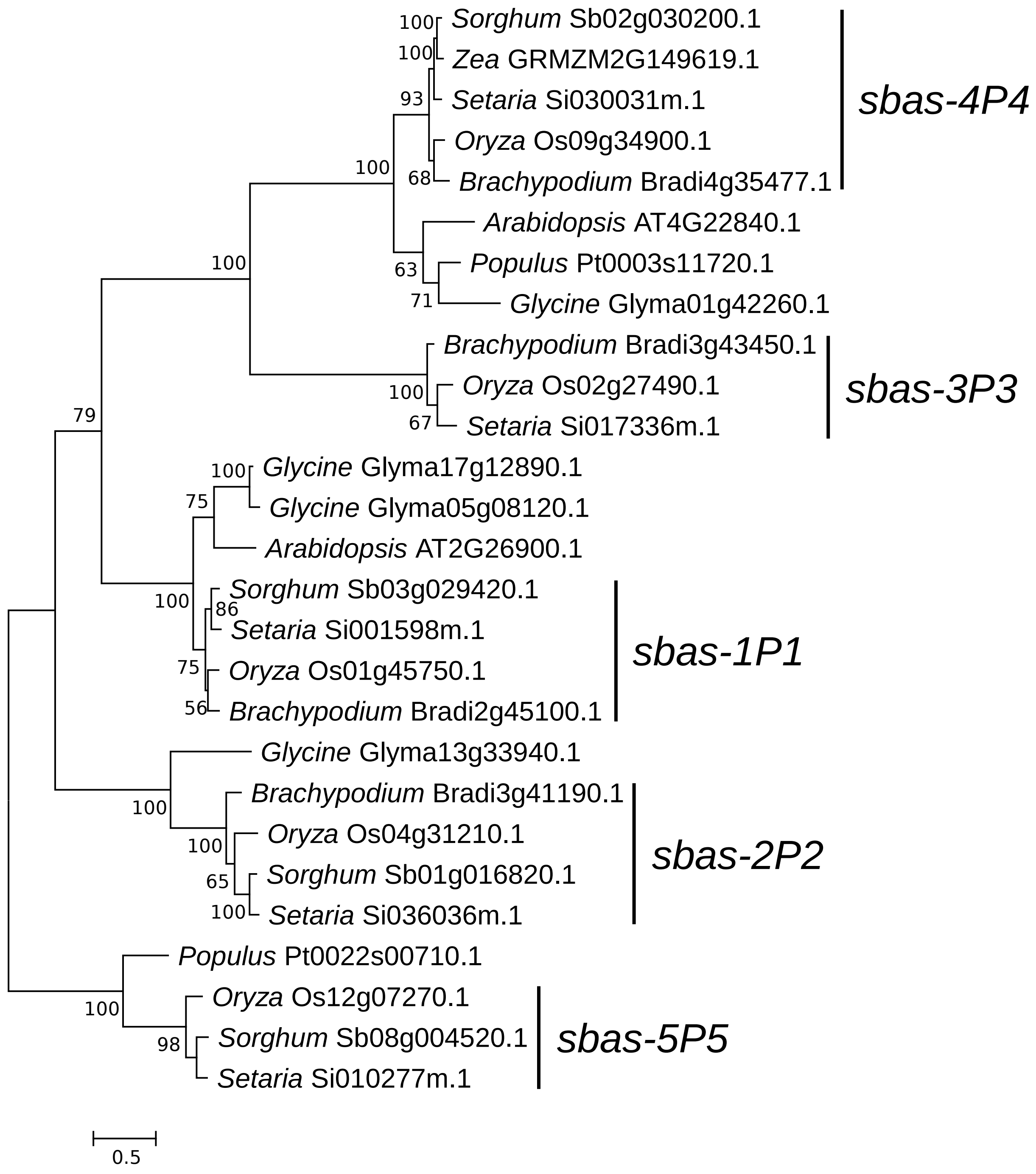

Sorghum Sb02g030200.1
Zea GRMZM2G149619.1
Setaria Si030031m.1
Oryza Os09g34900.1
Brachypodium Bradi4g35477.1
Arabidopsis AT4G22840.1
Populus Pt0003s11720.1
Glycine Glyma01g42260.1
Brachypodium Bradi3g43450.1
Oryza Os02g27490.1
Setaria Si017336m.1
Glycine Glyma17g12890.1
Glycine Glyma05g08120.1
Arabidopsis AT2G26900.1
Sorghum Sb03g029420.1
Setaria Si001598m.1
Oryza Os01g45750.1
Brachypodium Bradi2g45100.1
Glycine Glyma13g33940.1
Brachypodium Bradi3g41190.1
Oryza Os04g31210.1
Sorghum Sb01g016820.1
Setaria Si036036m.1
Populus Pt0022s00710.1
Oryza Os12g07270.1
Sorghum Sb08g004520.1
Setaria Si010277m.1
sbas-4P4
sbas-3P3
sbas-1P1
sbas-2P2
sbas-5P5
0.5

Fig. S3

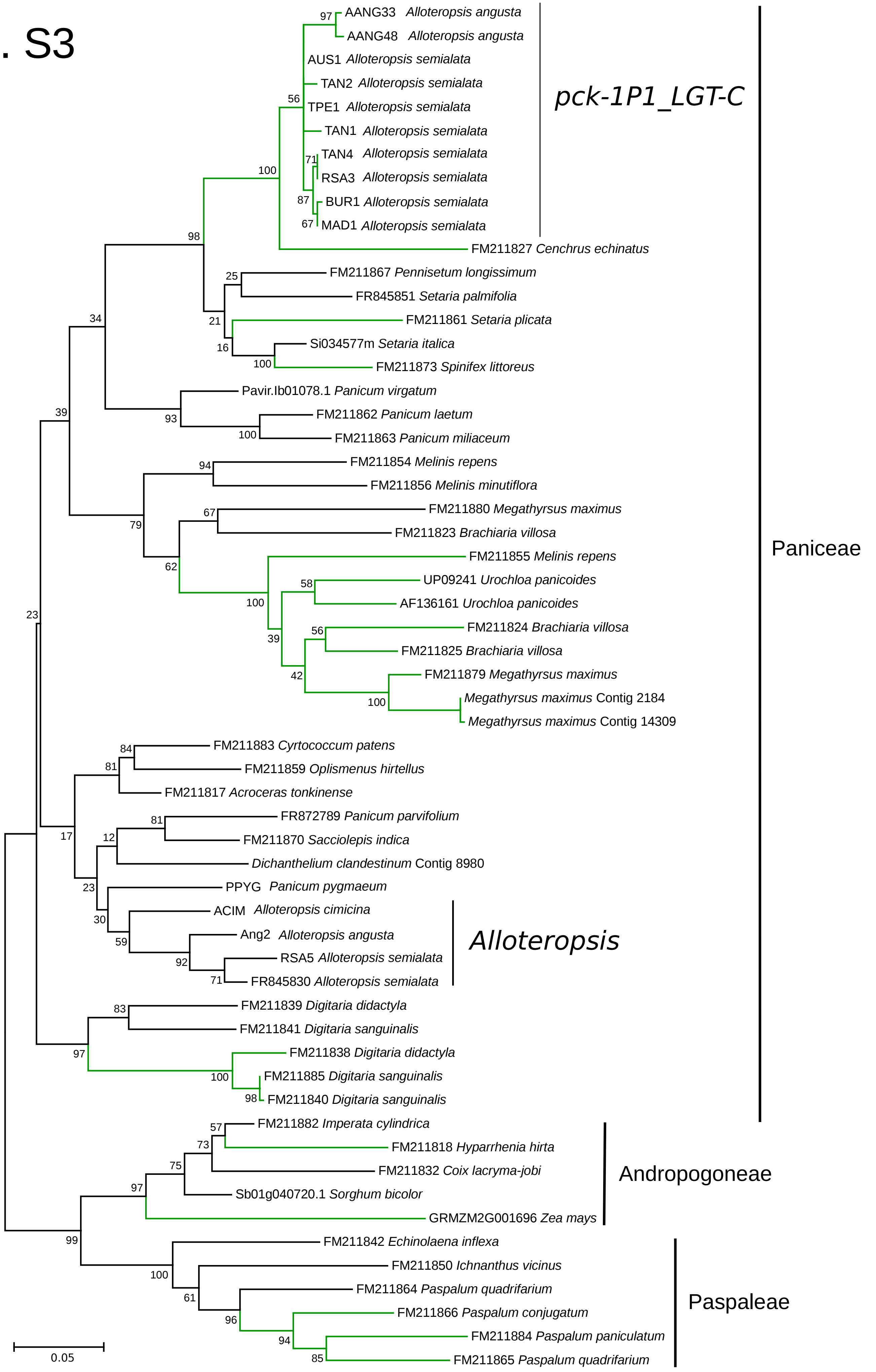

AANG33 Alloteropsis angusta
AANG48 Alloteropsis angusta
AUS1 Alloteropsis semialata
TAN2 Alloteropsis semialata
TPE1 Alloteropsis semialata
TAN1 Alloteropsis semialata
TAN4 Alloteropsis semialata
RSA3 Alloteropsis semialata
BUR1 Alloteropsis semialata
MAD1 Alloteropsis semialata
pck-1P1_LGT-C
FM211827 Cenchrus echinatus
FM211867 Pennisetum longissimum
FR845851 Setaria palmifolia
FM211861 Setaria plicata
Si034577m Setaria italica
FM211873 Spinifex littoreus
Pavir.Ib01078.1 Panicum virgatum
FM211862 Panicum laetum
FM211863 Panicum miliaceum
FM211854 Melinis repens
FM211856 Melinis minutiflora
FM211880 Megathyrsus maximus
FM211823 Brachiaria villosa
FM211855 Melinis repens
UP09241 Urochloa panicoides
AF136161 Urochloa panicoides
FM211824 Brachiaria villosa
FM211825 Brachiaria villosa
FM211879 Megathyrsus maximus
Megathyrsus maximus Contig 2184
Megathyrsus maximus Contig 14309
Paniceae
FM211883 Cyrtococcum patens
FM211859 Oplismenus hirtellus
FM211817 Acroceras tonkinense
FR872789 Panicum parvifolium
FM211870 Sacciolepis indica
Dichanthelium clandestinum Contig 8980
PPYG Panicum pygmaeum
ACIM Alloteropsis cimicina
Ang2 Alloteropsis angusta
RSA5 Alloteropsis semialata
FR845830 Alloteropsis semialata
Alloteropsis
FM211839 Digitaria didactyla
FM211841 Digitaria sanguinalis
FM211838 Digitaria didactyla
FM211885 Digitaria sanguinalis
FM211840 Digitaria sanguinalis
FM211882 Imperata cylindrica
FM211818 Hyparrhenia hirta
FM211832 Coix lacryma-jobi
Sb01g040720.1 Sorghum bicolor
GRMZM2G001696 Zea mays
Andropogoneae
FM211842 Echinolaena inflexa
FM211850 Ichnanthus vicinus
FM211864 Paspalum quadrifarium
FM211866 Paspalum conjugatum
FM211884 Paspalum paniculatum
FM211865 Paspalum quadrifarium
Paspaleae
0.05

Fig. S4

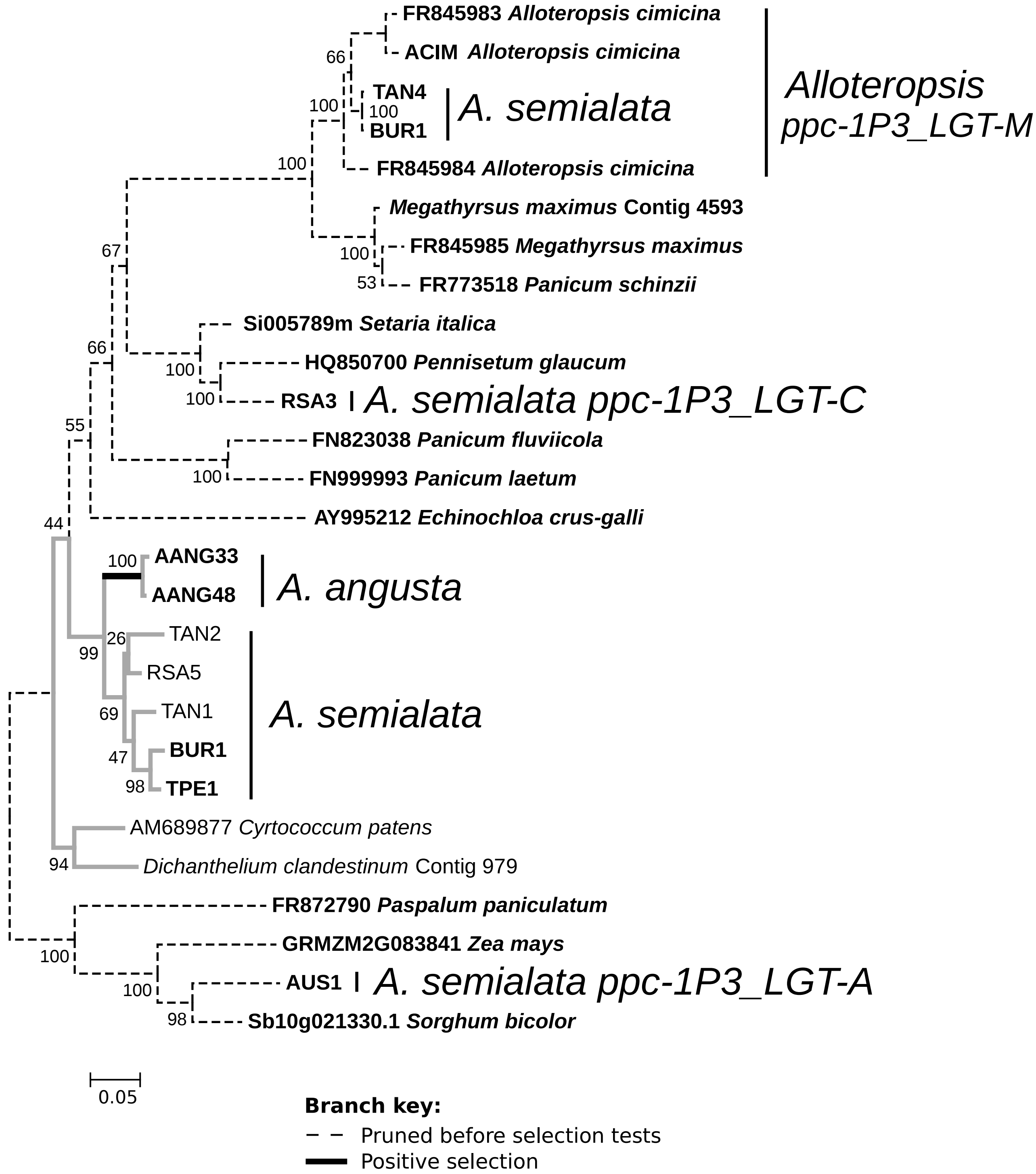
FR845983 Alloteropsis cimicina
ACIM Alloteropsis cimicina
TAN4
BUR1
A. semialata
FR845984 Alloteropsis cimicina
Alloteropsis
ppc-1P3_LGT-M
Megathyrsus maximus Contig 4593
FR845985 Megathyrsus maximus
FR773518 Panicum schinzii
Si005789m Setaria italica
HQ850700 Pennisetum glaucum
RSA3
A. semialata ppc-1P3_LGT-C
FN823038 Panicum fluviicola
FN999993 Panicum laetum
AY995212 Echinochloa crus-galli
AANG33
AANG48
A. angusta
TAN2
RSA5
TAN1
BUR1
TPE1
A. semialata
AM689877 Cyrtococcum patens
Dichanthelium clandestinum Contig 979
FR872790 Paspalum paniculatum
GRMZM2G083841 Zea mays
AUS1
A. semialata ppc-1P3_LGT-A
Sb10g021330.1 Sorghum bicolor
66
100
100
100
100
53
67
66
100
100
55
100
44
100
26
99
69
47
98
94
100
100
98
0.05
Branch key:
Pruned before selection tests
Positive selection

Fig. S5

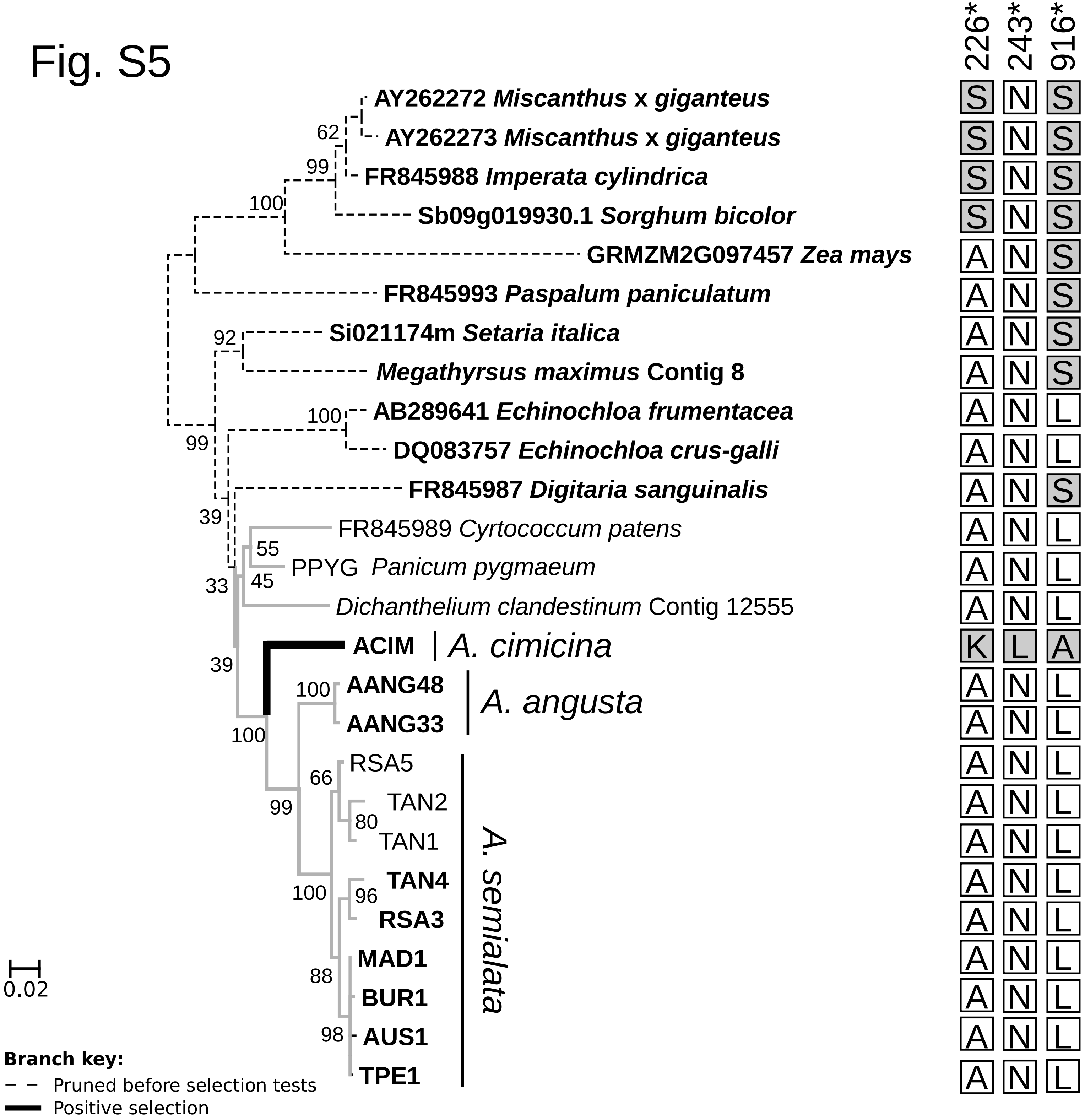
226*
243*
916*
AY262272 Miscanthus x giganteus
AY262273 Miscanthus x giganteus
FR845988 Imperata cylindrica
Sb09g019930.1 Sorghum bicolor
GRMZM2G097457 Zea mays
FR845993 Paspalum paniculatum
Si021174m Setaria italica
Megathyrsus maximus Contig 8
AB289641 Echinochloa frumentacea
DQ083757 Echinochloa crus-galli
FR845987 Digitaria sanguinalis
FR845989 Cyrtococcum patens
PPYG Panicum pygmaeum
Dichanthelium clandestinum Contig 12555
ACIM
A. cimicina
AANG48
AANG33
A. angusta
RSA5
TAN2
TAN1
TAN4
RSA3
MAD1
BUR1
AUS1
TPE1
A. semialata
62
99
100
92
100
99
39
55
45
33
39
100
100
66
80
99
96
100
88
98
0.02
Branch key:
Pruned before selection tests
Positive selection
S N S
S N S
S N S
S N S
A N S
A N S
A N S
A N S
A N L
A N L
A N S
A N L
A N L
A N L
K L A
A N L
A N L
A N L
A N L
A N L
A N L
A N L
A N L
A N L
A N L
A N L

Fig. S6

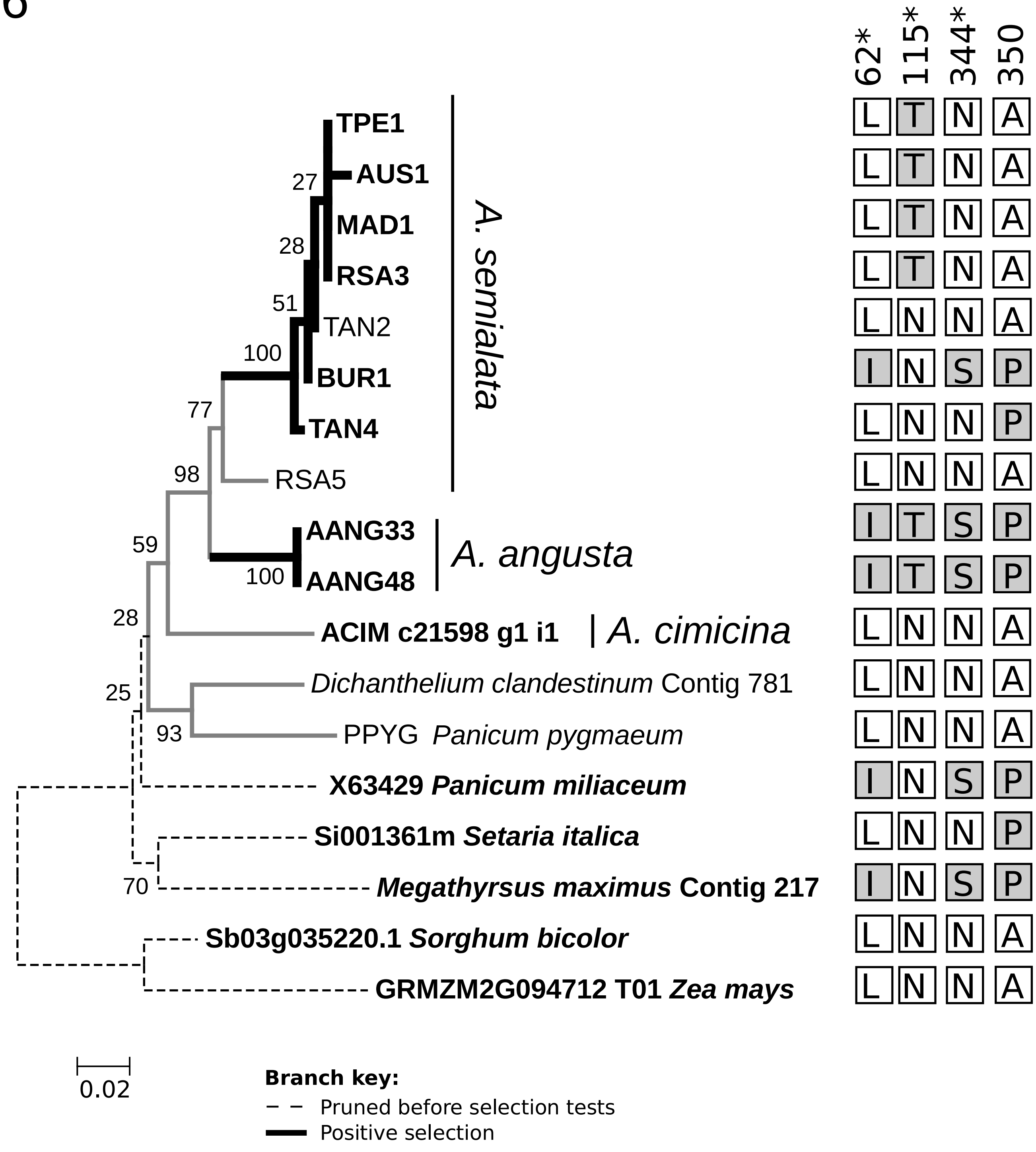
62*
115*
344*
350
TPE1
AUS1
MAD1
RSA3
TAN2
BUR1
TAN4
RSA5
A. semialata
AANG33
AANG48
A. angusta
ACIM c21598 g1 i1
A. cimicina
Dichanthelium clandestinum Contig 781
PPYG Panicum pygmaeum
X63429 Panicum miliaceum
Si001361m Setaria italica
Megathyrsus maximus Contig 217
Sb03g035220.1 Sorghum bicolor
GRMZM2G094712 T01 Zea mays
27
28
51
100
77
98
59
100
28
25
93
70
L T N A
L T N A
L T N A
L T N A
L N N A
I N S P
L N N P
L N N A
I T S P
I T S P
L N N A
L N N A
L N N A
I N S P
L N N P
I N S P
L N N A
L N N A
0.02
Branch key:
Pruned before selection tests
Positive selection
